# Steroid hormone antagonism affords vascular protection in a mouse model of vascular Ehlers-Danlos syndrome

**DOI:** 10.1172/jci.insight.198202

**Published:** 2026-04-28

**Authors:** Emily E. Juzwiak, Caitlin J. Bowen, Rhiannon Edwards, Leda Restrepo, Serena Lee, Cassie A. Parks, Anthony Zeng, Maya M. Black, Oscar E. Reyes Gaido, Emily E. Bramel, Dustin T. Shigaki, Michael A. Beer, Chiara Bellini, Harry C. Dietz, Elena Gallo MacFarlane

**Affiliations:** 1 McKusick-Nathans Department of Genetic Medicine, Johns Hopkins University, Baltimore, Maryland USA.; 2 Howard Hughes Medical Institute, Chevy Chase, Maryland, USA.; 3 Predoctoral training in Human Genetics and Molecular Biology, Johns Hopkins University, Baltimore, Maryland, USA.; 4 Department of Bioengineering, Northeastern University, Boston, Massachusetts, USA.; 5 Department of Dermatology, Brigham and Women’s Hospital, Boston, Massachusetts, USA.; 6 Department of Biomedical Engineering, Johns Hopkins University, Baltimore, Maryland, USA.; 7 Department of Surgery, Johns Hopkins University, Baltimore, Maryland, USA.

**Keywords:** Cell biology, Vascular biology, Extracellular matrix, Genetic diseases, Sex hormones

## Abstract

Aortic dissection or rupture is a leading cause of mortality in vascular Ehlers-Danlos syndrome (VEDS), a disorder caused by mutations in the *COL3A1* gene. *Col3a1^G938D/+^* mice recapitulate features of VEDS, including high risk of aortic rupture. As in people with VEDS, aortic risk in this model accelerates at the onset of puberty, especially in males. We identify developmentally regulated gene programs associated with this vulnerability and that are targeted by treatments that mitigate aortic risk. Both genetic and pharmacological inhibition of the androgen receptor (AR) eliminated survival differences between sexes, while treatment with a dual AR and mineralocorticoid receptor (MR) antagonist provided near-complete and durable protection in both sexes. Pathways targeted by dual AR/MR inhibition, including those related to extracellular matrix (ECM) organization and cell-ECM interactions, largely overlapped with those also modulated by isolated MR antagonism. Selective targeting of MR signaling emerged as an effective therapeutic strategy in both sexes that avoids sexual side effects in males.

## Abstract 2

Inhibition of androgen and mineralocorticoid receptor signaling in Vascular Ehlers-Danlos Syndrome improves survival in mice by upregulating extracellular matrix (ECM) organization and cell-ECM interactions.

## Introduction

Vascular Ehlers-Danlos syndrome (VEDS) is a heritable connective tissue disorder caused by heterozygous *COL3A1* mutations, which encode the pro-α1 chain of collagen III, a homotrimeric fibrillar collagen (1). Spontaneous aortic dissection or rupture of the thoracic or abdominal aorta are major mortality causes in patients (1, 2). Only 5%–10% of patients with VEDS present with aortic aneurysm before dissection/rupture, making clinical imaging ineffective at predicting catastrophic event location or timing (2). Although antihypertensive agents show modest effectiveness in some clinical trials, no disease-altering VEDS treatments currently exist (3, 4).

We previously described a VEDS mouse model harboring a heterozygous missense mutation (*Col3a1^G938D/+^*) causing glycine substitution in type III collagen’s triple helical domain, analogous to the most common patient pathogenic variant class (5). Both male and female VEDS mice die of spontaneous descending thoracic aorta rupture (with/without prior dissection), increasing in frequency between P30 and P60, and correlating with puberty onset and progression (5). Adverse aortic event acceleration during this window is more severe in male versus female VEDS mice, mirroring similarly heightened risk in young adult male patients with VEDS (5, 6).

In murine models, P30–P60 corresponds to rapid aortic wall growth and remodeling, which occurs in conjunction with the increase of hemodynamic parameters to adult levels and affects both extracellular matrix (ECM) composition and vascular smooth muscle cell (VSMC) differentiation (7). Circulating androgen concentration rises concurrently with these processes, starting around P30, peaking at P40, and leveling to steady-state adult levels at P60 (8). While androgen concentration increases occur in both sexes, absolute levels are higher in males throughout life (8). Our previous work indicates androgen-modulated pathways contribute to VEDS aortic vulnerability during this developmental transition, but their identity remains poorly understood (5). Like other steroid hormone receptors, the androgen receptor (AR) is a ligand-inducible transcription factor that remains cytoplasmic and inactive until testosterone or dihydrotestosterone binding (9).

Here, we identify developmentally regulated biological processes associating with increased aortic rupture vulnerability and modulated by therapies mitigating rupture risk in VEDS mice. We provide genetic evidence that androgen signaling is the major contributor to sexual dimorphism in arterial dissection/rupture risk in this model. We also show mineralocorticoid receptor–modulated (MR-modulated) pathways partly overlap with AR-modulated pathways, and that MR antagonism represents a sex-independent therapeutic option for mitigating aortic rupture in VEDS.

## Results

### Increased risk of aortic rupture in VEDS mice correlates with postnatal downregulation of synthetic programs and VSMC differentiation.

**Figure 1:**
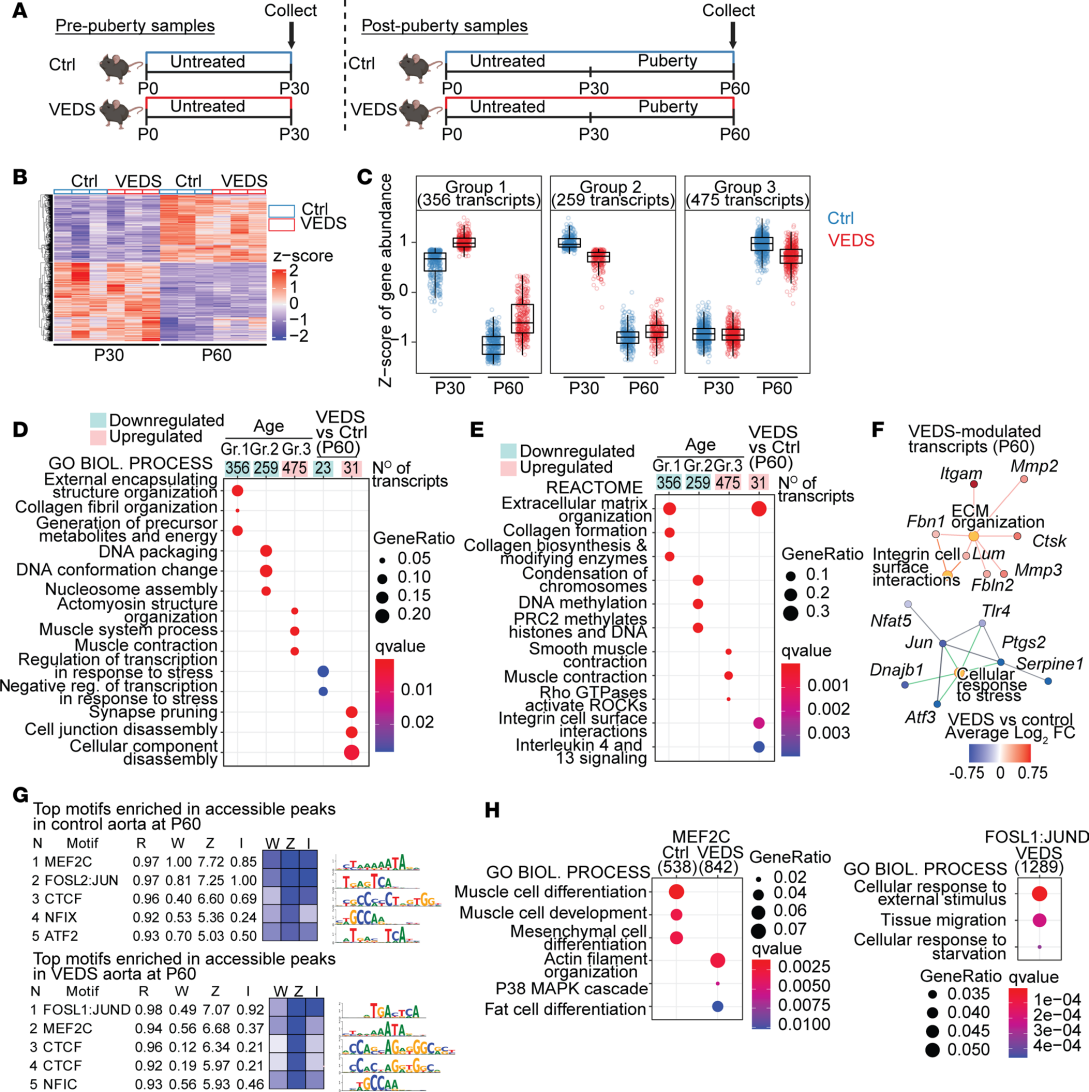
Increased risk of aortic rupture in VEDS mice correlates with postnatal downregulation of synthetic programs and VSMC differentiation. (**A**) Diagram of experimental conditions for bulk RNA-seq of descending thoracic aorta from untreated control *Col3a1^+/+^* (Ctrl) and *Col3a1^G938D/+^* (VEDS) mice. (**B**) Heatmap of differentially expressed transcripts between P30 (prepuberty) and P60 (postpuberty) in Ctrl and VEDS samples (FDR ≤ 0.05, abs Log_2_FC ≥ 0.25). (**C**) Divisive clustering identified 3 major groups of coregulated genes. (**D** and **E**) GO biological process (**D**) and Reactome (**E**) enrichment for coregulated transcripts and genotype-dependent genes at P60. (**F**) STRING networks of enriched terms and transcripts in VEDS versus control aorta at P60. Node colors indicate expression in VEDS: red (upregulated), blue (downregulated). (**G**) Heatmap and logos of top motifs enriched in accessible chromatin regions (ATAC-seq) in control or VEDS aorta, determined by gkm-PWM algorithm. Heatmap shows Redundancy (R), Weight (W), *z* score (Z), and Importance (I). (**H**) GO biological process enrichment for genes near indicated transcription factor binding sites. For **D**, **E**, and **H**: GeneRatio = proportion of genes in pathway/term; *q*-value = FDR-adjusted *P*-value (cutoff *P* ≤ 0.05); top 3 pathways/terms shown.

We previously showed that adolescent male *Col3a1^G938D/+^* mice have increased aortic rupture risk relative to females and that androgen inhibitors are necessary to maintain hydralazine’s protection (initiated at birth) beyond P30–P45 (5, 10). To understand this developmental vulnerability, reproduced in current cohorts (Supplemental Figure 1, A and B; supplemental material available online with this article; https://doi.org/10.1172/jci.insight.198202DS1), we examined transcriptional changes in the descending thoracic aorta of control (*Col3a1^+/+^*) and VEDS (*Col3a1^G938D/+^*) male mice between P30 (prepuberty) and P60 (postpuberty) using bulk RNA-seq (Figure 1A). Despite robust aortic phenotypes in VEDS mice, most transcriptional changes were age- rather than genotype-driven (|Log_2_FC| ≥ 0.25, FDR ≤ 0.05) (Figure 1B and Supplemental Table 1), separating into 3 coregulated groups by divisive clustering (Figure 1C).

Age-dependent coregulated transcripts and genotype-specific alterations were examined for GO biological process and Reactome pathway enrichment (Figure 1, D and E). Group 1 transcripts, downregulated from P30 to P60 but less so in VEDS aorta (Figure 1C), were enriched for ECM synthesis/remodeling (*Col1a2*, *Col3a1*, *Col4a3*, *Eln*, *Loxl2*, *Mmp14*, *Mmp2*, *Serpinh1*), growth factor receptors promoting VSMC proliferation/migration (*Pdgfra*), metabolic enzymes for energy production/cellular respiration (*Acacb*, *Acadvl*, *Aco1*, *Aco2*, *Atp5o*, *Slc25a51*, *Uqcr10*), and metabolic signaling modulators (*Adipor2*, *Ppara*) (Figure 1, D and E, and Supplemental Table 1). Group 2 transcripts, related to cell proliferation (histone genes, cell cycle modulators), were also downregulated between P30 and P60 (Figure 1, C–E, and Supplemental Table 1). Group 3 transcripts were developmentally upregulated regardless of genotype, though slightly decreased in VEDS versus controls (Figure 1C), including actomyosin contractile components (*Lmod1*, *Myh11*, *Myl9*, *Nebl*, *Rock1*, *Rock2*, *Tpm1*), cell-substrate adhesion factors (integrins *Itga1*, *Itga3*, *Itga5*, *Itgb1*, *Itgb5*), and VSMC differentiation signals (*Tgfb3*, *Tgfbr1*, *Rbpms*, *Rbpms2*) (Figure 1, D and E, and Supplemental Table 1). Few transcripts differed between VEDS and control aorta; these included upregulated ECM components/remodeling enzymes (*Fbln2*, *Fbn1*, *Igfbp6*, *Lum*, *Mmp2*, *Ctsk*) and downregulated stress response/tissue repair factors (*Jun*, *Ptgs2*, *Tlr4*, *Dnajb1*, *Atf3*) (Figure 1F and Supplemental Table 1).

ATAC-seq chromatin profiling of control and VEDS aortic nuclei at P60 showed concordant developmental transitions and similar transcription factor activity between genotypes (Supplemental Figure 2, A and B, and Supplemental Table 1). Analysis via the gapped k-mer position weight matrixes (gkmPWM) algorithm identified MEF2C and AP-1–related transcription factors (JUN, JUND, FOSL1, FOSL2) motifs as most enriched in accessible chromatin regions (Figure 1G and Supplemental Figure 2B). However, gene loci near predicted MEF2C binding sites were enriched for VSMC differentiation factors in controls (*Hes1*, *Jag1*, *Rock1*, *Itga8*, *Rbpms2*) whereas gene loci near MEF2C were enriched for actin filament organization modulators (*Arhgap6*, *Gsn*, *Svil*, *Synpo*) and stress-activated p38 kinase signaling (*Gadd45b*, *Gadd45g*, *Mapkapk2*, *Phlpp1*, *Zfp36*) in VEDS aorta (Figure 1H and Supplemental Table 1). Gene loci with FOSL1:JUND motifs in VEDS aorta (but not FOSL2:JUN in controls) were enriched for stress/starvation response pathways (*Foxo1*, *Foxo3*, *Prkag2*, *Prkag3*, *Cdkn1a*) (Figure 1H and Supplemental Table 1). These data suggest increased aortic vulnerability in VEDS associates with physiological downregulation of synthetic/proliferative programs, with VEDS-specific transcriptional alterations at P60 potentially representing compensation or pathogenic deviation.

### Coadministration of hydralazine with androgen inhibitors induces upregulation of transcripts promoting ECM deposition.

**Figure 2:**
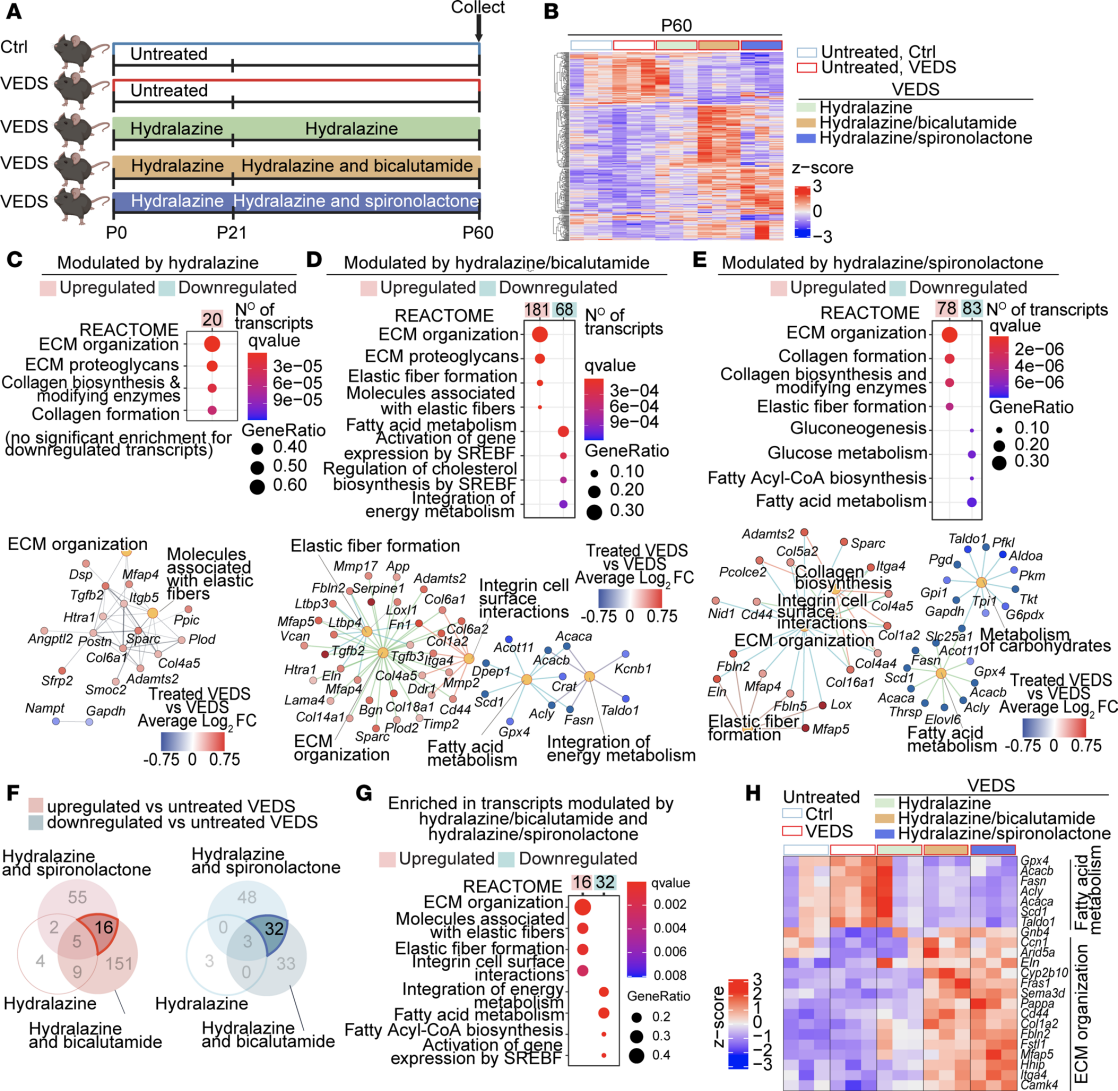
Coadministration of hydralazine with androgen inhibitors induces upregulation of transcripts promoting ECM deposition. (**A**) Diagram of experimental conditions for bulk RNA-seq of descending thoracic aorta from treated and untreated *Col3a1^G938D/+^* (VEDS) mice and untreated *Col3a1^+/+^* controls (Ctrl). (**B**) Heatmap of differentially expressed transcripts across all samples (FDR ≤ 0.05). (**C**–**E**) Reactome pathway enrichment and STRING networks of transcripts modulated by hydralazine (**C**), hydralazine/bicalutamide (**D**), and hydralazine/spironolactone (**E**). Network nodes colored by expression change in treated versus untreated VEDS: red (upregulated), blue (downregulated). (**F**) Venn diagram of transcripts modulated by each treatment versus untreated VEDS aorta. (**G**) Pathway enrichment for transcripts concordantly modulated by both hydralazine/bicalutamide and hydralazine/spironolactone. (**H**) Heatmap of concordantly modulated transcripts. For **C**–**E**, and **G**: GeneRatio = proportion of genes in pathway/term; *q* value = FDR-adjusted *P* value (*P* ≤ 0.05); top 4 pathways shown.

To examine how developmentally regulated transcriptional changes were affected by treatments protecting VEDS mice from aortic rupture, we analyzed aortas collected at P30 and P60 from control and VEDS mice treated with hydralazine since birth, plus VEDS mice receiving hydralazine with either bicalutamide (a potent androgen antagonist) (11–13) or spironolactone (a dual androgen/MR antagonist) (14, 15) starting at P21 (Figure 2A).

Pair-wise comparisons between treated and untreated VEDS aortic samples identified transcripts modulated by hydralazine alone and by cotreatment with bicalutamide or spironolactone (Figure 2B and Supplemental Table 2). All treatments upregulated ECM synthesis–related transcripts, including elastogenesis and collagen deposition, though hydralazine alone modulated fewer transcripts (Figure 2, C–E). Hydralazine/bicalutamide and hydralazine/spironolactone cotreatments also downregulated energy metabolism transcripts, including fatty acid metabolism (Figure 2, D and E). A core set of 48 transcripts was modulated in an AR-dependent manner by hydralazine/bicalutamide and hydralazine/spironolactone, but not hydralazine alone (Figure 2F and Supplemental Table 2), largely corresponding to age- rather than genotype-dependent changes (Figure 2, G and H, and Supplemental Table 2). AR inhibition counteracted aging effects by upregulating transcripts for ECM components involved in collagen and elastin deposition, including *Col1a2*, *Eln*, *Fras1*, *Itga4*, *Mfap5*, and *Fstl1*, which encodes a matrix protein recently shown to protect against acute aortic dissection (16) (Figure 2, G and H, and Supplemental Table 2). Other age-dependent processes were either unaffected (downregulation of pro-proliferative pathways) or enhanced (downregulation of fatty acid biosynthesis) by AR antagonism (Figure 2, G and H, and Supplemental Table 2). This transcriptional analysis suggested AR inhibition efficacy associated not with restoring VEDS transcriptional programs to age-matched control levels, but with increasing ECM-related transcript expression beyond healthy control levels.

### Isolated genetic and pharmacologic androgen antagonism improves survival in male VEDS mice.

Pathway overlap in transcripts modulated by hydralazine/bicalutamide or hydralazine/spironolactone cotreatment, and the modest effect of hydralazine alone, suggested isolated AR antagonism may suffice to improve VEDS mouse survival.

**Figure 3:**
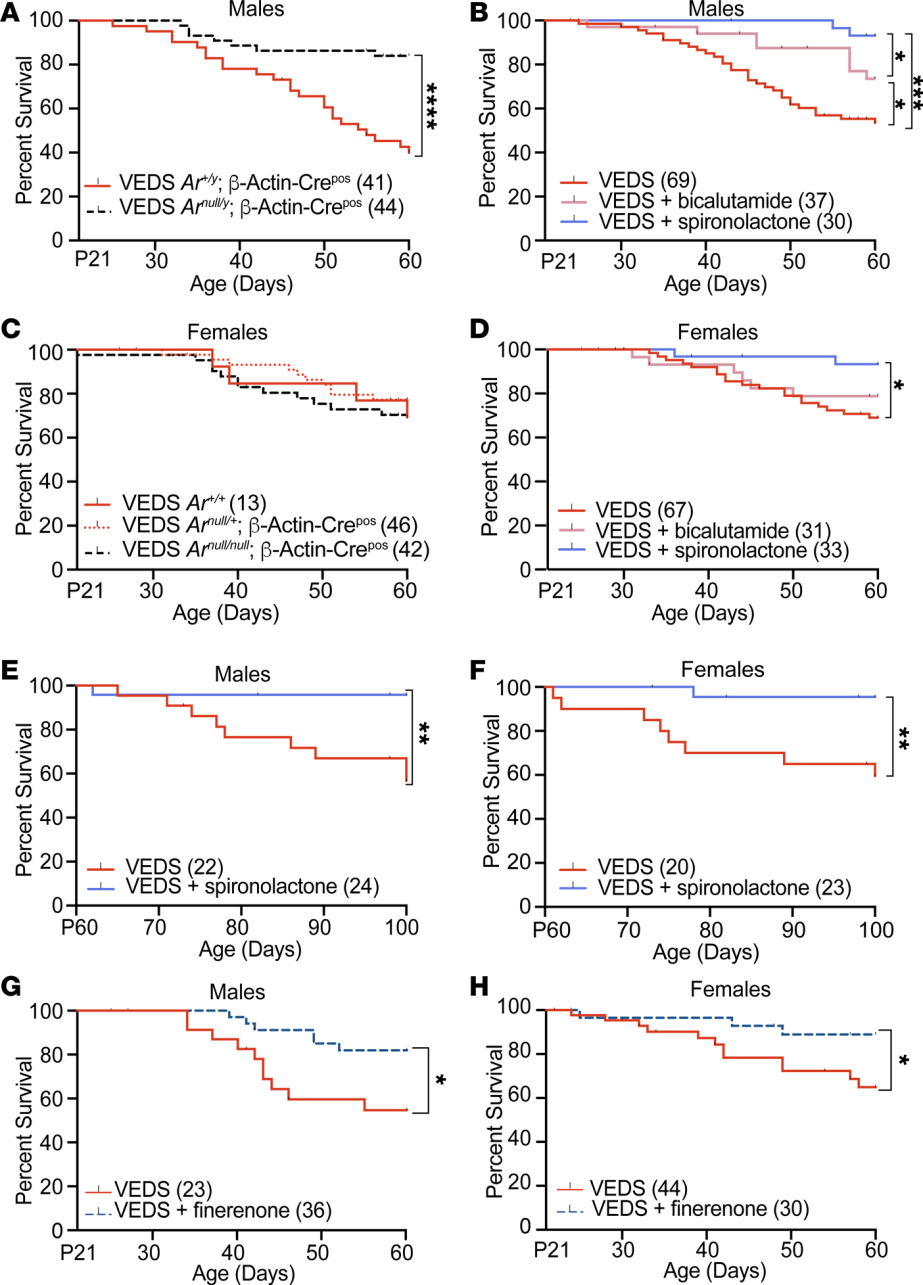
AR antagonism eliminates sexual dimorphism in survival, while MR antagonism protects VEDS mice of either sex from aortic rupture. (**A**) Kaplan-Meier survival curve comparing control *Ar^+/y^*; β-actin–Cre^+^ (*n* = 41) and VEDS *Ar^null/y^*; β-actin–Cre^+^ (*n* = 44) male mice. (**B**) Kaplan-Meier survival curve comparing untreated male VEDS mice (*n* = 69) and bicalutamide- (*n* = 37) or spironolactone- (*n* = 30) treated (P21-P60) male VEDS mice. (**C**) Kaplan-Meier survival curve comparing VEDS *Ar^+/+^* (*n* = 13), VEDS *Ar^null/+^*; β-actin–Cre^+^ (*n* = 46), and VEDS *Ar^null/null^*; β-actin– Cre^+^ (n = 42) female mice. (**D**) Kaplan-Meier survival curve comparing untreated female VEDS mice (*n* = 67) and bicalutamide- (*n* = 33) or spironolactone-treated (*n* = 33) (P21–P60) female VEDS mice treated. (**E**) Kaplan-Meier survival curve comparing untreated male VEDS mice (*n* = 22) and male VEDS mice treated with spironolactone starting at P60 (*n* = 24). (**F**) Kaplan-Meier survival curve comparing untreated female VEDS mice (*n* = 20) to female VEDS mice treated with spironolactone starting at P60 (*n* = 23). (**G**) Kaplan-Meier survival curve comparing untreated male VEDS mice (*n* = 23) and finerenone-treated (P21–P60) male VEDS mice (*n* = 36). (**H**) Kaplan-Meier survival curve comparing untreated female VEDS mice (*n* = 44), and finerenone-treated (P21–P60) female VEDS mice (*n* = 30). *P* values refer to Log-rank Mantel-Cox analysis. For all panels, *P ≤ 0.05, **P ≤ 0.01, ***P ≤ 0.001, and ****P ≤ 0.0001.

To test this, we examined how bicalutamide treatment or genetic Ar inactivation affected aortic rupture risk in VEDS mice. Mice carrying a conditional *Ar^flox^* allele (X chromosome) (17) were crossed to VEDS mice expressing β-actin–Cre recombinase, generating globally *Ar*-deficient VEDS mice. *Ar* expression, increased in postpubertal aortic walls of both sexes, was effectively abrogated by Cre-mediated deletion in male *Ar^null/y^* and female *Ar^null/null^* mice (Supplemental Figure 3). In males, global *Ar* deletion (VEDS *Ar^null/y^*) improved survival from 40% to 84%, and pharmacological AR inhibition with bicalutamide improved male VEDS survival from 53% to 74% versus untreated controls (Figure 3, A and B). Unlike hydralazine/bicalutamide cotreatment, which benefits both sexes, neither genetic *Ar* inactivation nor bicalutamide alone improved female VEDS survival (Figure 3, C and D). No residual sex-dependent survival differences existed between bicalutamide-treated VEDS males and untreated VEDS females (*P* = 0.53) or between *Ar*-deficient VEDS males and VEDS females (*P* = 0.29). Although tissue fragility precludes measurement of blood pressure by tail cuff plethysmography in VEDS mice, assessment in *Col3a1^+/+^* control mice showed that *Ar* disruption did not affect male blood pressure but slightly increased it in females (Supplemental Figure 4A). Bicalutamide affected neither *Ar* expression nor blood pressure in either sex (Supplemental Figures 3 and 4). These data indicate that, while AR antagonism protects males, residual risk persists in VEDS mice of both sexes, suggesting the existence of additional AR-independent modulators of aortic rupture risk.

### Pharmacologic MR antagonism improves survival in both male and female VEDS mice.

The elimination of sexual dimorphism by pharmacologic or genetic AR inhibition suggested androgen signaling accounted for most survival differences between male and female VEDS mice. However, bicalutamide has clinical limitations due to sexual side effects in males (11–13, 18). We thus examined the effect of isolated spironolactone treatment, an FDA-approved AR inhibitor with fewer sexual side effects (14, 15). Unlike bicalutamide, spironolactone improved survival in both male and female VEDS mice, resulting in near-complete P60 protection (54% to 93% in males; 69%–97% in females) (Figure 3, B and D). Spironolactone reduced aortic rupture risk even when initiated after puberty (P60), improving P100 survival from 57% to 95% in males and 60% to 95% in females (Figure 3, E and F). Like bicalutamide, spironolactone did not affect *Ar* expression or blood pressure (Supplemental Figures 3 and 4).

The greater protection by spironolactone versus bicalutamide or genetic *Ar* inhibition led us to hypothesize that the MR, also inhibited by spironolactone, may be a VEDS therapeutic target (15, 16). To isolate MR inhibition effects, we examined how finerenone, a selective MR antagonist (19) affected VEDS mouse survival. Finerenone improved survival in both males (55% to 82%) and females (65% to 89%) (Figure 3, G and H), without changing blood pressure (Supplemental Figure 4). While finerenone did not affect *Ar* expression in either sex (Supplemental Figure 3), *Nr3c2* (encoding MR) expression increased in finerenone-treated female aortas but remained unchanged in other groups (Supplemental Figure 5). MR inhibition’s beneficial effect was independent of testosterone modulation, as circulating testosterone remained unaffected by AR, AR/MR, or MR inhibition (*P* = 0.25 for males; *P* = 0.66 for females) (Supplemental Figure 6). These data suggest that MR antagonism modulates aortic rupture risk in both sexes through an AR-independent mechanism.

### AR/MR antagonism increases expression of transcripts promoting ECM deposition and aerobic respiration in multiple cell types.

**Figure 4:**
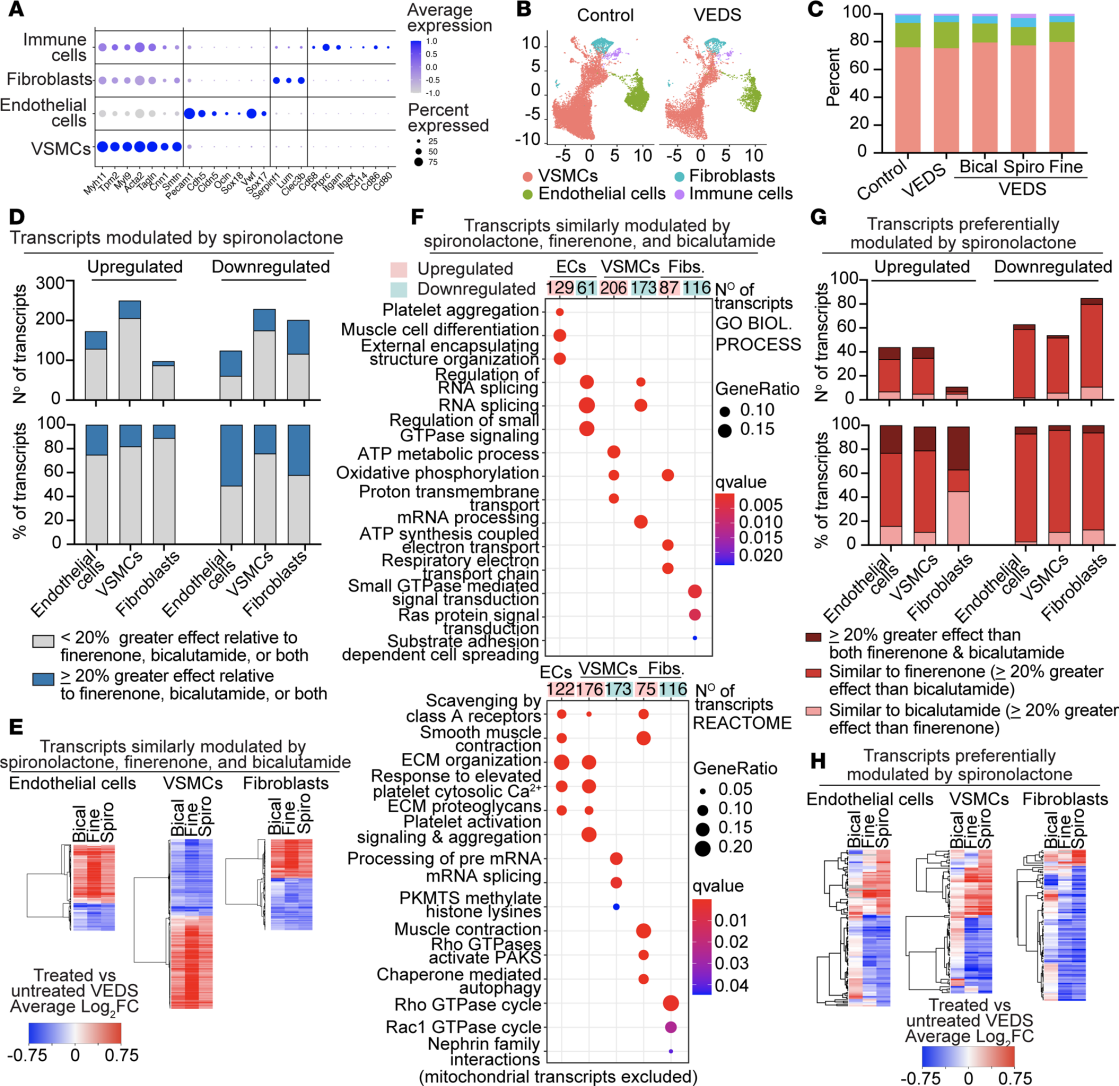
AR/MR inhibition upregulates transcripts promoting ECM deposition and aerobic respiration, and downregulates transcripts related to Rho/Rac signaling and alternative mRNA splicing in multiple cell-types. (**A**) Dot plot of cluster-defining transcripts for cluster identity. Dot color intensity indicates scaled average expression; dot size shows percentage of nuclei expressing each transcript per cluster. (**B**) Unsupervised clustering (Louvain resolution 0.1) identifies 4 major aortic cell populations: vascular smooth muscle cells (VSMCs), endothelial cells, fibroblasts, and immune cells, stratified by genotype (control versus VEDS). (**C**) Stacked bar plot showing nuclei proportion per cluster and no significant differences between treatment groups (2-way ANOVA). (**D**) Numbers and percentages of transcripts similarly modulated by spironolactone, finerenone, and bicalutamide or preferentially by spironolactone versus one or both other treatments (|Log_2_FC| ≥ 0.25, *P*_adj_ ≤ 0.05). (**E**) Heatmap and hierarchical clustering of transcripts similarly modulated by isolated or dual AR/MR antagonism. Expression in spironolactone (Spiro)-, finerenone- (Fine-), and bicalutamide-treated (Bical-treated) samples shown relative to untreated VEDS aorta. (**F**) GO biological process (ribosomal transcripts excluded) and Reactome (ribosomal and mitochondrial transcripts excluded) pathway enrichment for transcripts modulated by isolated or dual AR/MR antagonism. GeneRatio = proportion of transcripts in term set; *q* value = FDR-adjusted *P* value (*P* ≤ 0.05). (**G**) Numbers and percentages of transcripts preferentially modulated by spironolactone versus finerenone, bicalutamide, or both in major aortic cell types. (**H**) Heatmap of transcripts from **G**. For **E** and **H**: red = upregulated; blue = downregulated versus untreated VEDS.

To overcome bulk RNA analysis limitations, potentially confounded by different cell type transcriptional profiles, and examine cell-specific transcriptional changes from isolated AR and MR inhibition (without hydralazine), we performed single-nucleus RNA-seq (snRNA-seq) on descending thoracic aortas of VEDS mice treated with spironolactone, bicalutamide, or finerenone from P21 to P60 (*n* = 3/group), plus untreated controls and VEDS mice. Clusters corresponding to VSMCs, endothelial cells, fibroblasts, and immune cells were identified using previously described cluster-defining transcripts (Figure 4A) (20), with no genotype- or treatment-specific cell populations or over-/underrepresentation detected between groups (Figure 4, B and C). Immune cells were excluded due to inadequate recovered nuclei depth and lack of inflammatory involvement in VEDS (5).

To identify protection mechanisms common to AR and MR antagonism versus those specific to dual AR/MR inhibition, we focused on transcripts modulated by spironolactone (|Log_2_ FC| ≥ 0.25 versus untreated VEDS; *P*_adj_ ≤ 0.05), given its superior aortic rupture prevention efficacy and dual AR/MR inhibitor activity. Spironolactone-modulated transcripts were subdivided into those comparably modulated across all treatments versus those preferentially modulated by spironolactone. We reasoned that dual AR/MR antagonism’s enhanced survival benefit could stem from qualitative (which transcripts are modulated), quantitative (transcriptional change level), and combinatorial effects (concurrent AR/MR target modulation in 1 or more cell types).

Transcripts preferentially modulated by spironolactone were identified as those for which the effect of treatment was, approximately, 20% or more that of bicalutamide, finerenone, or both (corresponding to ~0.25 difference between spironolactone versus untreated VEDS Log_2_ FC and other treatments versus untreated VEDS). Using this threshold, ~60%–80% of spironolactone-modulated transcripts were comparably modulated by finerenone and bicalutamide across all 3 cell types (Figure 4, D and E, and Supplemental Table 3). Enriched pathways among comparably upregulated transcripts included ECM deposition and aerobic glycolysis (Figure 4F, Supplemental Figure 7, and Supplemental Table 3).

Upregulated transcripts encoded diverse ECM components (*Bgn*, *Col1a1*, *Sparc*, *Fn1*, *Fbln5*, *Mfap4*) and integrin β1 (*Itgb1*), partially overlapping those age-dependently downregulated by bulk RNA-seq (Supplemental Figure 7 and Supplemental Table 3). Downregulated transcripts across all cell types were related to Rho/Rac1 network modulation, including RhoGEFs and RhoGAPs (Supplemental Figure 7 and Supplemental Table 3).

Although selected contractile factors were upregulated in VSMCs (*Acta2*, *Tpm2*, *Myl6*, *Myl9*), transcripts associated with differentiated, contractile VSMC phenotype (*Mylk*, *Myocd*, *Smtn*, *Pdlim7*, *Lmod1*, *Ncoa3*, *Prdm16*) (21–23) were similarly downregulated by all treatments, consistent with a partially “modulated” phenotype in VEDS VSMCs (Supplemental Figure 7 and Supplemental Table 3). Transcripts for factors involved in alternative mRNA processing, an integrin-modulated process associating with VSMC phenotypic transition (24), were also downregulated by treatment in VSMCs (25) (Figure 4F, Supplemental Figure 7, and Supplemental Table 3). In fibroblasts, all treatments promoted contractile protein expression (*Acta2*, *Tpm2*, *Myl6*, *Myl9*) and downregulated negative TGF-β signaling modulators, including *Smurf2* (25), *Ldlrad4/C18ORF1* (26), and *Mycbp2* (27) (Supplemental Figure 7F). Surprisingly, treatment effects on endothelial cells resembled those in VSMCs, including upregulation of contractile proteins and ECM components, and downregulation of alternative splicing modulators (Figure 4F, Supplemental Figure 7, and Supplemental Table 3).

### Overlapping biological processes are modulated by isolated or concomitant AR/MR inhibition with spironolactone.

We also examined the 20%–40% of transcripts preferentially modulated by spironolactone versus finerenone, bicalutamide, or both in each cell type (Figure 4D). For most transcripts in this group, spironolactone’s effect mirrored finerenone’s (Figure 4, G and H, and Supplemental Figures 8–10). Fewer transcripts were similarly modulated by bicalutamide and spironolactone, or exclusively by spironolactone (Figure 4, G and H, and Supplemental Figures 8–10). Pathway enrichment for transcripts preferentially up- or downregulated by spironolactone alone overlapped with those modulated by isolated AR or MR inhibition (Figure 4, Supplemental Figures 8–10, and Supplemental Table 3).

**Figure 5:**
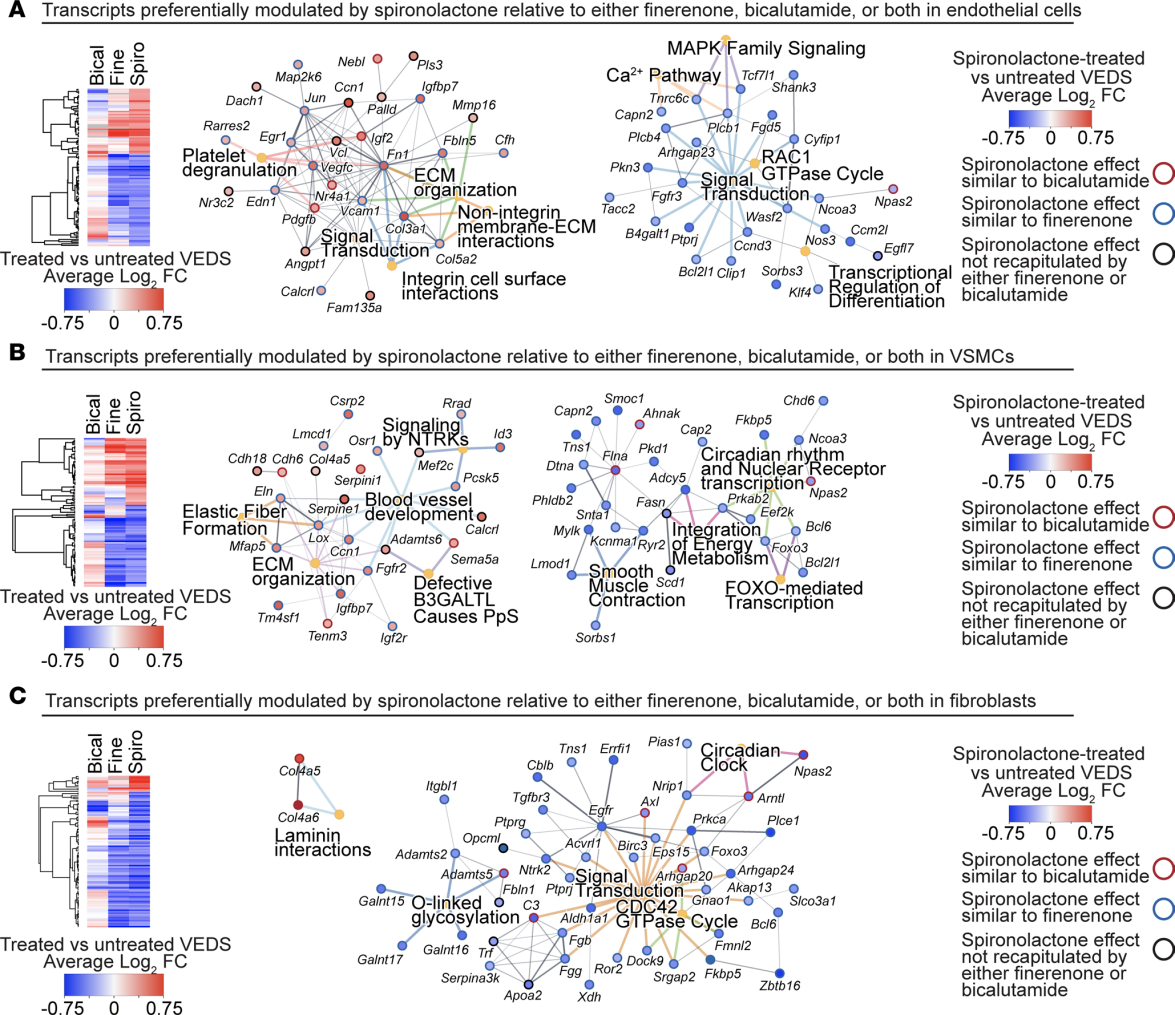
Pathways enriched within transcripts preferentially modulated by spironolactone overlap with those modulated in a comparable manner by isolated AR or MR antagonisms. (**A**–**C**) Heatmaps and STRING networks of enriched terms for transcripts preferentially modulated by spironolactone versus finerenone, bicalutamide, or both in endothelial cells (**A**), VSMCs (**B**), and fibroblasts (**C**). Heatmaps show expression in spironolactone- (Spiro-), finerenone- (Fine-,) and bicalutamide-treated (Bical-treated) samples relative to untreated VEDS aorta. Network nodes colored by expression change in spironolactone-treated versus untreated VEDS: red (upregulated), blue (downregulated). Node borders indicate whether spironolactone effect is recapitulated by bicalutamide (dark red), finerenone (blue), or neither (black). Magnified heatmaps are shown in Supplemental Figures 8–10.

Combinatorial effects of dual AR/MR antagonism in endothelial cells included upregulation of both high-affinity adrenomedullin (AM) receptor subunits, encoded by *Calcrl* (upregulated by spironolactone) and *Ramp2* (upregulated by all treatments), which promote endothelial expression of tight junction, adherens junction, and basement membrane molecules (28) (Figure 5A, Supplemental Figures 7 and 8, and Supplemental Table 3). Several transcriptional changes suggested stabilized cell-cell junctions and cell-matrix adhesion, including increased expression of angiopoietin-1 (*Angpt1*) (29) and vinculin (*Vcl)* (30) (spironolactone only), collagen V (*Col5a2*) (31) and fibronectin (*Fn1*) (spironolactone and finerenone), Nebulette (*Nebl*) (32) (spironolactone and bicalutamide) (Figure 5A, Supplemental Figure 8, and Supplemental Table 3), integrin β1 (*Itgb1*), claudin-5 (*Cldn5*) (33), and basement membrane collagens (*Col4a1*, *Col4a5*) (all treatments) (Supplemental Figure 7 and Supplemental Table 3). ECM deposition and barrier integrity stabilization were exemplified by downregulation of calpain-2 (*Capn2*) (34), phospholipase C β1 and β4 (*Plcb1*, *Plcb4*) (35, 36), and protein kinase N3 (*Pkn3*) (37) (spironolactone and finerenone); VE-statin (*Egfl7*) (38) (spironolactone only); proline-rich tyrosine kinase 2 (*Ptk2*) (39); and membrane associated ring-CH-type finger 3 (*March3*) (40) (all treatments) (Figure 5A, Supplemental Figures 7 and 8, and Supplemental Table 3).

In VSMCs, spironolactone and finerenone, but not bicalutamide, upregulated transcripts encoding elastin (*Eln*), lysyl oxidase (*Lox*), and microfibril associated protein 5 (*Mfap5*), whereas fibulin-5 (*Fbln5*) was also upregulated by bicalutamide, all indicating upregulated elastogenic gene programs (Figure 5B, Supplemental Figures 7 and 9, and Supplemental Table 3) (41). Other matrix components specifically upregulated by spironolactone and finerenone, but not bicalutamide, included insulin-like growth factor binding protein 7 (*Igfbp7*) (42), cellular communication network factor (*Ccn1*) (41), LIM and cysteine rich domains 1 (*Lmcd1*) (43), and basement membrane collagen (*Col4a5*) (Figure 5B and Supplemental Figure 8). Basement membrane collagens (*Col4a1*, *Col4a5*) were also among few transcripts more pronouncedly upregulated by spironolactone versus other treatments in adventitial fibroblasts (Figure 5C and Supplemental Figure 10).

### Protection from rupture correlates with induction of a protective transcriptional signature in VSMCs.

**Figure 6:**
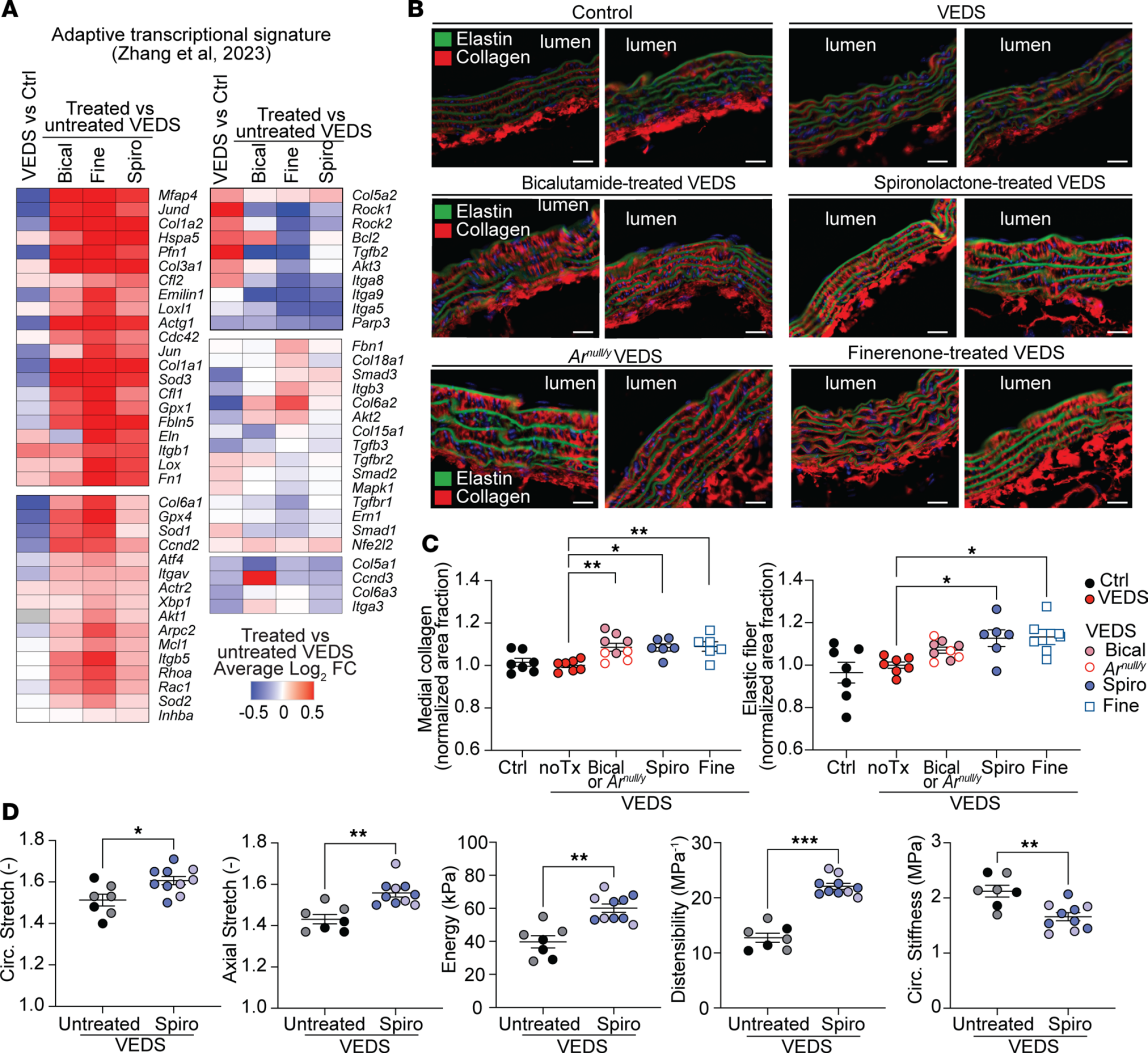
AR/MR antagonism upregulates adaptive transcriptional signature in VSMCs, increases medial collagen and elastin content, and alters the passive mechanical properties of the aorta in VEDS mice. (**A**) Heatmap of transcripts previously associated with adaptive response to mechanical stress in aneurysmal aorta (44). Expression is shown in VSMCs from VEDS versus control aorta, and spironolactone (Spiro)-, finerenone (Fine)-, and bicalutamide (Bical)-treated versus untreated VEDS mice. Red = upregulated; blue = downregulated. (**B**) Representative aortic sections from descending aorta of untreated control, untreated VEDS, and VEDS males treated with spironolactone, finerenone, or bicalutamide or with genetic *Ar* inactivation. Images show elastin (Alexa Fluor 633 hydrazide, green) and collagen (tdTomato-CNA35, red). Scale bar: 20 μm. (**C**) Quantification of medial collagen and elastic fiber content (normalized to the average value for untreated VEDS) in the descending thoracic aorta of male untreated control (*n* = 7) and VEDS (*n* = 7) mice, spironolactone- (Spiro-) (*n* = 6) or finerenone-treated (Fine-treated) (*n* = 6) treated VEDS mice, VEDS mice following genetic (*Ar^null/y^*, *n* = 4), or pharmacological (Bical, *n* = 5) androgen receptor inhibition. Each symbol represents an individual replicate; *P* value refers to 1-way ANOVA with Šídák post hoc test. (**D**) Passive biomechanical properties of descending thoracic aortas from untreated (*n* = 7) and spironolactone-treated (*n* = 10) VEDS mice under physiological pressure loads. Each symbol represents an individual replicate; light shading represents females. *P* value refers to Mann-Whitney *U* test. *P ≤ 0.05, **P ≤ 0.01, ***P ≤ 0.001.

Transcriptional alterations from AR/MR antagonism in VEDS VSMCs partly resembled the adaptive aortic stress response induced by yes-associated protein (YAP) activation (44). Of 80 “adaptive transcripts” defined by Zhang et al. (44), 64 were detected in our dataset; 8 (*Itgb1*, *Col5a2*, *Bcl2*, *Akt3*, *Tgfb2*, *Rock1*, *Rock2*, *Itga8*) were upregulated in VEDS VSMCs versus untreated controls, independently of treatment (Log_2_ FC ≥ 0.20, *P*_adj_ ≤ 0.05) (Figure 6A and Supplemental Table 3). AR/MR antagonists further upregulated integrin β1 (*Itgb1*), collagen (*Col1a1*, *Col3a1*, *Col1a2*, *Lox*), elastic fiber formation molecules (*Mfap4*, *Eln*, *Fn1*, *Emilin1*, *Fbln5*), and oxidative damage-protective enzymes (*Gpx1, Sod3*) (Figure 6A). However, transcripts encoding integrin α9 (*Itga9*), α8 (*Itga8*), and α5 (*Itga5*) were downregulated by spironolactone, while others (*Smad3*, *Itgb3*, *Col6a2*, *Fbn1*, *Bcl2*, *Tgfbr2*, *Tgfbr1*, *Akt3*, *Mapk1*, *Smad2*, *Tgfb2*, *Akt2*, *Smad1*, *Col15a1*, *Ern1*, *Tgfb3*, *Col18a1*) were unaffected (44) (Figure 6A and Supplemental Table 3), suggesting encoded factors may be unnecessary or hindering for protection. Since chronic synthetic pathway activation can provoke VSMC transition toward a deleterious “degradative” phenotype (45), we examined how spironolactone, finerenone, and bicalutamide modulated transcripts previously characterized as defining “contractile,” “synthetic,” “phagocyte-like” (lysosome/protein degradation organelles), “activated,” and “immune-like” VSMC phenotypes (45). Protective treatments upregulated transcripts associated with both contractile and synthetic phenotypes plus those related to proteasome-dependent protein degradation and autophagy; conversely, only *B2m* (encoding MHC class I–associated beta-2-microglobulin) was upregulated among “activated” VSMC phenotype transcripts (*Mmp2*, *Mmp14*, *Mmp16*, *Vcam1*, *B2m*, *Spp1*, *Tnfrs11b*, *Cx3cl11*), and “immune-like” transcripts were undetected in VSMCs (Supplemental Figure 11A). Both bulk-RNA seq and snRNA-seq showed correlation between treatment effectiveness and inhibition of lipogenic enzyme transcripts (*Fasn*, *Scd1*, *Acly*) (Figures 2 and 5). Decreased fatty acid synthase protein levels were confirmed in bulk aortic protein lysates following AR and MR inhibition (Supplemental Figure 11B).

### AR/MR inhibition modulates ECM composition and passive biomechanical properties of the aorta.

Increased elastogenesis and collagen synthesis gene expression suggested that upregulation of these pathways may compensate for collagen III deficiency and contribute to AR/MR inhibition’s protective effect. We thus examined aortas of *Ar*-deficient and bicalutamide-, spironolactone-, or finerenone-treated VEDS mice for collagen and elastin content. Histological assessment with picrosirius red staining (PSR) showed expected adventitial collagen content reduction in VEDS aortas, as previously observed (5), but revealed no treatment-dependent changes (Supplemental Figure 12). However, examination of medial collagen and elastic fiber content with pancollagen and elastin-binding probes (46, 47) revealed increases in relative collagen content following AR antagonism and both collagen and elastin following MR inhibition in male samples (Figure 6, B and C). Although similar elastic fiber content increases following spironolactone treatment were detected in female samples, other parameters were unaffected by treatments, possibly due to small sample size (Supplemental Figure 13).

To determine whether observed increases in relative collagen and elastin content associated with changes in aortic biomechanics, we examined passive mechanical responses of aortic tissues from spironolactone-treated and untreated VEDS mice (Figure 6D). Analysis showed spironolactone treatment rendered VEDS aortic tissues more deformable in both circumferential and axial directions across a broad, physiologically relevant stretch range, requiring greater stretch to achieve given stress levels (Supplemental Figure 14A). Accordingly, at representative systolic pressure, spironolactone-treated aortic tissues extended to higher biaxial stretch (Figure 6D) while maintaining stress levels comparable with nontreated mice (Supplemental Figure 14B). Under these loading conditions, spironolactone treatment selectively reduced circumferential tissue stiffness (Figure 6D) while preserving axial stiffness (Supplemental Figure 14B). This behavior conferred greater elastic energy storage during systole, indicating enhanced aortic capacity to function as a pressure reservoir augmenting diastolic blood flow (Figure 6D). Consistent with tissue behavior and preserved wall thickness (Supplemental Figure 14B), spironolactone-treated mice further exhibited increased cyclic distensibility, reflecting reduced structural aortic stiffness as a conduit (Figure 6D).

These data suggest that AR/MR antagonism promotes a partial “synthetic” VSMC phenotype resulting in increased ECM deposition that, together with additional effects on endothelial cell barrier function and adventitial fibroblasts, improves mechanical function and protects VEDS mice from aortic rupture, counteracting physiological downregulation of ECM synthetic pathways occurring at postnatal aortic development completion.

## Discussion

In this study, we examined transcriptional signatures associated with increased aortic rupture frequency in *Col3a1^G938D/+^* VEDS mice during the P30–P60 window, and risk reduction following AR and/or MR signaling interventions. Phenotypic improvement with AR and MR inhibition correlated with partial reversal of transcriptional alterations accompanying postnatal aortic transition from proliferative/synthetic to quiescent/contractile phenotype, resulting in upregulated elastogenesis and collagen deposition programs. These changes were detected both by bulk RNA-seq in VEDS aortas treated with AR/MR inhibitors plus hydralazine and by snRNA-seq in VSMCs from VEDS aortas treated with spironolactone, finerenone and, to a lesser extent, bicalutamide.

Phenotypically, these transcriptional alterations associated with increased medial collagen content following AR and/or MR antagonist treatment, while increased medial elastin required MR inhibition. In all cases, microstructural alterations exceeded control aorta basal levels, suggesting compensatory response against defective collagen III assembly. This altered matrix balance contextualizes AR/MR antagonism’s biomechanical effects in VEDS aorta following spironolactone treatment. Functionally, noted microstructural changes are consistent with greater elastin-mediated load bearing and delayed collagen recruitment under physiological loading. Tissue- and vessel-level signatures include enhanced elastic energy storage and increased cyclic distensibility, indicating greater pulsatile pressure accommodation capacity. These features align with clinical observations of improved arterial elasticity in hypertensive patients receiving spironolactone (48).

Beyond modulating VSMC phenotype, AR/MR antagonism may reduce aortic rupture risk by affecting endothelial cells and fibroblasts. AR/MR antagonism-induced transcriptional alterations in endothelial cells suggested stabilized cell-cell junctions and increased basement membrane component and adhesion molecule synthesis. Functional outcomes of these alterations remain to be elucidated, but the role of endothelial cell dysfunction in aortic dissection/rupture was recently documented in other hereditary aortopathy models (49). In fibroblasts, AR/MR inhibition promoted a transcriptional profile consistent with induction of a myofibroblast-like phenotype. Despite these transcriptional alterations, adventitial collagen content was not altered by AR/MR inhibition, suggesting any protective effect may depend on other matrix components or mechanisms.

Although most transcripts were comparably modulated by isolated or dual AR/MR inhibition, 20%–40% showed preferential spironolactone modulation versus finerenone, bicalutamide, or both. Pathways enriched within these transcripts generally overlapped with those enriched among transcripts modulated by isolated AR or MR antagonism. Dual AR/MR inhibition’s increased therapeutic benefit may, thus, result from both enhanced protective biological process modulation and combinatorial AR/MR target modulation in one or more cell types.

Spironolactone- and finerenone-regulated transcript overlap across multiple cell types exceeded bicalutamide-sensitive transcript overlap, and both drugs improved VEDS mouse survival sex-independently. These data indicate MR signaling is a targetable pathway for aortic risk modulation in VEDS, with important translational implications. Bicalutamide’s clinical utility as androgen signaling inhibitor is hampered by male adverse effects, including gynecomastia, loss of libido, and erectile dysfunction (11–13, 18). Conversely, spironolactone reaches therapeutic AR blockade levels with lower male gynecomastia rates while affording MR inhibition protection (19, 50). While spironolactone could be considered for vascular protection in patients with VEDS of both sexes, FDA-approved MR antagonists such as finerenone or eplerenone may offer alternatives avoiding potential male sexual side effects and would, based on our preclinical data, potentially benefit both sexes. While functional provocations are necessary to probe specific pathway contributions to AR- and/or MR inhibition therapeutic efficacy, our results suggest MR antagonism warrants translational exploration as a VEDS therapeutic, particularly in males. Future studies may investigate whether MR antagonism reduces elastolysis and/or promotes elastin repair, mechanisms that may underlie the observed improvement in elastic fiber structure and vessel elasticity. Additionally, determining whether systemic administration of spironolactone and/or finerenone affects elastic fiber content in skin and other organs could provide accessible biomarkers for translational studies in patients with VEDS.

## Methods

### Sex as a biological variable.

Both sexes were used to identify sex-dependent or sex-independent treatment effects. Sex is indicated in figure legends. Males were used for bulk RNA-seq and snRNA-seq due to more severe untreated phenotype.

### Mouse husbandry.

*Col3a1^G938D/+^* mice were generated as described (5). Mice carrying the *Ar^flox^* allele were gifted by Sheng Wu, Temple University (17). Global *Ar* null animals were obtained by crossing *Ar^flox^* mice with β-actin–Cre mice (The Jackson Laboratory, #033984). All mice were maintained on C57BL/6J background (The Jackson Laboratory, #00064), housed in individually ventilated cages with ad libitum access to standard chow and water in the Miller Animal Research Facility at Johns Hopkins University. Contemporaneous untreated cohorts were used as controls for survival studies. Survival was not different between *Col3a1^G938D/+^* *Ar^+/+^*; β-actin–Cre^+^ mice and *Col3a1^G938D/+^* *Ar^+/+^*; β-actin–Cre^neg^ mice (not shown); therefore, these groups were combined as controls.

### Delivery of medication.

Treatments were formulated assuming consumption of 2–4 g food/day and 5 mL water/day by mice with a 20–25 g weight. Hydralazine (American Health Packaging, NDC 68084-447-01) was dissolved in water (32 mg/kg/day from birth). The following drugs were mixed with powdered food (LabDiet 5001): bicalutamide (Northstar Rx, NDC 16714-816-02) at 0.625 mg/g (50 mg/kg/day); spironolactone (Accord Healthcare, NDC 16729 227 01) at 1.25 mg/g (100 mg/kg/day); and finerenone (Bayer Healthcare Pharmaceuticals, NDC 50419-540 or Selleck Chemicals, S8841) at 0.05 mg/g (5 mg/kg/day).

### Tissue collection and processing for bulk RNA-seq.

Prepuberty samples were collected at P30; post-puberty at P60–P61, with descending thoracic aortas harvested between 11 a.m. to 1 p.m. Mice were euthanized by isoflurane inhalation, the abdominal inferior vena cava was transected, and aortas were perfused with 5 mL PBS through the right ventricle. Descending thoracic aortic segments (left subclavian to diaphragm) were collected, cleaned of perivascular adipose tissue, placed in screw-cap tubes with 700 μL TRIzol (Thermo Fisher, 15596018) plus four to six 3 mm zirconium beads (OPS Diagnostics, BAWS 3000-300-23), flash frozen in liquid nitrogen, and stored at –80°C.

Within treatment groups (*n* = 3), aortas were randomized in batches of 10 (1 per group), homogenized using FastPrep-24 5G (MP Biomedicals; 5 cycles: 4.5 m/s, 30 seconds, 300-second pause; Lysing Matrix A, 1 mg), then 200 μL chloroform (Fisher Scientific, C298) was added, shaken 15 seconds, and incubated 3 minutes at room temperature. After centrifugation (12,000 × *g*, 15 minutes, 4°C), RNA was extracted with RNeasy Kit (Qiagen, 74104) using 350 μL ice-cold 70% ethanol and 350 μL Wash Buffer I, treated with DNase I (70 μL buffer RDD + 10 μL DNase I; RNase-Free DNase set, Qiagen, 79254; 15 minutes at room temperature), eluted in 100 μL nuclease-free water, and stored at –80°C. Samples were submitted to Sidney Kimmel Cancer Center Genomics Core for QC (Agilent 2100 BioAnalyzer), cDNA synthesis, and library generation with TruSeq Stranded Total RNA Library Kit (Illumina, 20020598), and then sequenced on NovaSeq6000 S4 (150 bp PE), targeting 50,000 reads/sample. CASAVA 1.8.4 converted BCL to FASTQ files; RSEM-1.3.0 aligned to mm39 genome using “rsem-calculate-expression” with the following options: star, star-gzipped-read-file, calc-ci, star-output-genome-bam, paired-end, forward-prob 0.

### Bulk RNA-seq analysis in RNAseqChef.

RNAseqChef (51) was used to perform integrative transcriptome analysis. “Multi DEG” function with divisive clustering was applied to untreated control and VEDS samples at P30 and P60, filtering for transcripts modulated between timepoints (|Log_2_ FC| ≥ 0.25, FDR ≤ 0.05). Pair-wise EBseq function identified differentially expressed transcripts between control and VEDS at P60 and those modulated by treatments (FDR ≤ 0.05).

### Gene enrichment and network visualization.

Overrepresentation analysis was performed with the RNAseqChef gene enrichment visualizer or Cytoscape/STRING. Heatmaps for bulk RNA-seq used z-scores from normalized counts; snRNA-seq heatmaps used Log_2_ FC (Morpheus, Broad Institute). Custom networks were generated using Cytoscape/STRING, and nodes custom-filled based on differential expression between samples.

### ATAC-seq data generation, peak calling and annotation.

Nuclei from aortic tissue (P60, *n* = 2 per genotype) were prepared using Chromium Next GEM Single Cell ATAC Kit v2 (10X Genomics, PN-1000406) reagents and workflow, sequenced on NovaSeq 6000 (Illumina) at Johns Hopkins GRCF, and aligned to mm10. Accessible regions were called with MACS2 (v2.2.7.1) in narrow-peak mode with barcode-aware duplicate removal and Tn5 cut-site correction; ENCODE mm10 blacklist regions were excluded. Peaks were annotated with ChIPseeker (v1.40.0) using TxDb.Mmusculus.UCSC.mm10.knownGene and org.Mm.eg.db, with promoter-proximal regions defined as ± 3 kb of transcription start sites (TSS). Library-level enrichment around TSS was quantified from CPM normalized bigWigs, and FRiP was computed as the fraction of fragments overlapping consensus peaks.

### Sequence modeling and motif characterization.

Gkm–support vector machines (gkm-SVMs) (52) were trained on 300 bp windows centered on ATAC peak summits using standard parameters (l = 11, k = 7). GC- and length-matched negatives were sampled from nonpeak genomic regions. PWMs were derived with gkmPWM (53) in lasso mode (gkmPWMlasso), fitting known PWM libraries with L1-penalized regression to approximate the same weights and rank motifs, providing coefficients indicating each motif’s unique contribution after accounting for redundancy. Best-matching motif entries in the MEME library are listed based on Redundancy (R): maximum Pearson correlation to any other PWM learned in the same run; Weight (W): model contribution score assigned by gkmPWM; Z-score (Z): standardized mean of the highest-weighted gkm; Importance (I): relative increase in model error when removing that PWM.

### Motif scanning and transcription factor binding sites (TFBS) calling.

The top 2 transcription factors (by I score) were mapped to 300-bp sequences with mapTF (53), using the same (l, k) parameters as the trained gkm-SVM models. High-confidence sites in each accessible region were retained using prespecified thresholds (average k-mer posterior probability ≥ 0.90 and model motif correlation ≥ 0.80). Peaks were assigned to genes by nearest-TSS linkage on mm10 using a genome-indexed nearest-neighbor search and labeled promoter-proximal if distance ≤ 3 kb, and distal if otherwise.

### Transcript and protein quantification.

Aortic tissue was homogenized using FastPrep-24 5G (MP Biomedicals) (5 cycles, 4.5 m/s, 30 seconds, 300-second pause, Lysing Matrix A at 1 mg) as described above. RNA was prepared as for bulk RNA-seq, while protein extracts were obtained using the Protein Extraction Kit (Full Moon Biosystems, EXT020) with PhosSTOP tablets (Roche, 04906837001) and cOmplete Mini EDTA-free Protease Inhibitor Cocktail (Roche, 04693159001), and then flash frozen. RNA was quantified by Nanodrop ND-1000 (Thermo Fisher) and 10 ng RNA was used for cDNA synthesis with TaqMan High-Capacity cDNA Reverse Transcription reagents (Applied Biosystems, 4368814). Quantitative PCR was performed in duplicate with TaqMan Universal PCR Master Mix (Applied Biosystems, 4304437) on QuantStudio 7 (Applied Biosystems). TaqMan probes used were: Mm03951035 (*Ar*), Mm01241596 (*Nr3c2*), and Mm99999915 (*Gapdh*). Relative abundance was normalized to *Gapdh* then to untreated adult sex-matched WT controls using the 2^–ΔΔCT^ method. Prepuberty samples were collected P29–P31, postpuberty (adult) samples P58-64.

Immunoblotting (P58–P64 mice) was performed with standard protocols using LI-COR reagents and the LI-COR Odyssey imaging system (LI-COR Biosciences). Primary antibodies directed against β-Actin (8H10D10) (Cell Signaling Technology, 3700) and FASN (Cell Signaling Technology, 3189) were incubated in blocking buffer (LI-COR BioSciences, 927-60001) overnight. IRDye-conjugated secondary antibodies IRDye-680RD goat anti-mouse (LI-COR BioSciences, L926-68070) and IRDye-800CW goat anti-rabbit (LI-COR Biosciences, 926-32211) were used to detect signal for β-actin and FASN, respectively.

### Blood pressure measurement.

Blood pressure was measured by tail cuff plethysmography using Visitech BP-2000, as previously described (5). All blood pressure measurements refer to control mice, as weak adherence of the skin to the tail in VEDS mice precludes assessment via tail cuff plethysmography. Each data point represents average of 10–15 systolic or 3–15 diastolic measurements per mouse. Sex- and age-matched controls were measured concurrently.

### Histological assessment.

Thoracic aortas were perfused, fixed in 4% PFA (EMS, 50-980-487) overnight, transferred to 70% ethanol, and paraffin embedded. Longitudinal sections (5 μm) were stained with PSR (StatLab, KTPSRPT) and imaged at 20× (3 areas per sample, Nikon Eclipse E400). Total collagen was quantified as red channel area/total tissue area (54) using Fiji 1.54 (55).

Sections were also prepared for medial collagen and elastin examination using tdTomato-CNA35 probe (Addgene, plasmid 61606) and Alexa Fluor 633–hydrazide (Invitrogen, A30634), respectively, according to established protocols (46, 47, 56). Entire longitudinal sections were imaged at 20× using Leica Mica Microhub with instant computational clearing; medial collagen and elastin were quantified as area/total medial area using Fiji 1.54. A blinded operator masked adipose, debris, and folds prior to all quantification.

### Measurement of circulating testosterone by ELISA.

Whole blood was collected terminally from the abdominal inferior vena cava (P58–P64 mice, collected between 10 a.m. to 1 p.m.) using 23 G butterfly needle and 3 mL syringe into BD microtainer (catalog 365967). Blood sat 30–60 minutes at room temperature, then was centrifuged (2,000*g*, 20 minutes, 4°C), flash frozen, and stored at –80°C. For each sample, 100 μL of serum was examined with Cayman Chemicals testosterone ELISA kit (Cayman Chemicals, 582701) after diethyl ether extraction per manufacturer’s instructions. Each sample was assayed in triplicate. Outliers (≥3 SD from the mean) were removed before interpolation, and interpolated data Log_10_ transformed for normality.

### Single-nuclei isolation.

Descending thoracic aortas (P59-61, no visible aneurysm/dissection) were flushed with chilled DPBS (Thermo Fisher, J67670-K2), flash frozen at –154°C, and processed using Chromium Nuclear Isolation Kit (10X Genomics, 1000494). Three aortas per treatment group (5 groups) were randomized in batches (1 per group, *n* = 5 per batch), cross-hatched on ice in glass petri dishes, placed in Lysis Buffer (9–12 minutes on ice), and manually homogenized with pestle (10 twists, release, repeat 4×). Samples were resuspended in 1 mL Wash and Resuspension Buffer containing RNase inhibitor, kept on ice until all batch samples were ready, and then centrifuged at 500*g* for 10 minutes at 4°C, resuspended in 50 μL Wash and Resuspension Buffer, and submitted to Johns Hopkins Single Cell & Transcriptomics Core for QC, cDNA, and library synthesis.

### snRNA-seq, alignment, and matrix generation.

Nuclei counts and metrics were determined using Cell Countess II (Thermo Fisher) with DAPI staining. A maximum volume of 86.4 μL/sample was used to target up to 10,000 nuclei. Nuclei were combined with RT reagents, loaded onto 10X Next GEM Chip M with 3’ HT gel beads (10X Genomics, cat 1000281), and processed using the NextGEM protocol on 10X Chromium X to create GEMs (gel beads in emulsion). Approximately 180 μL of emulsion was retrieved, split into 2 wells, incubated (45 minutes at 53°C, 5 minutes at 85°C, cool to 4°C), generating barcoded cDNA from each nucleus. GEMs were broken using Chromium Recovery Agent (10X Genomics, 220016); cDNA was cleaned using MyOne SILANE beads (10X Genomics, 2000048) and amplified for 11 cycles (3 minutes at 98°C; 11 cycles: 15 seconds at 98°C, 20 seconds at 63°C, 1 minute at 72°C; then 1 minute at 72°C, cool to 4°C). Samples were cleaned using 0.6X SPRIselect beads (Beckman Coulter, B23317); 20 μL amplified cDNA was carried into library preparation. Fragmentation, end repair, and A-tailing were completed (5 minutes at 32°C, 30 minutes at 65°C, cool to 4°C); samples were cleaned using double-sided size selection (0.6X, 0.8X) with SPRIselect beads, followed by adaptor ligation (15 minutes at 20°C, cool to 4°C), 0.8X cleanup, and amplification with PCR using unique i7 and i5 index sequences. Libraries underwent final double-sided size selection cleanup (0.6X, 0.8X) with SPRIselect beads, were quantified using Qubit (Thermo Fisher, Q33231) and KAPA library quantification qPCR kit (Roche, 07960140001), and they were sequenced on Illumina NovaSeq 6000 using v1.5 kits (Illumina, 20028319), targeting 50,000 reads/cell at read lengths of 28 (R1), 10 (i7), 10 (i5), 91 (R2). Demultiplexing and FASTQ generation was completed using Illumina’s BaseSpace software; alignment was completed using 10X Genomics CellRanger software (version cellranger-7.1.0) count and aggr functions, aligning to reference transcriptome refdata-gex-mm10-2020-A.

### snRNA-seq analysis.

Analysis was carried out using R (version 4.3.1) and Seurat (version 4.4.0). In total, 127,386 nuclei were identified by CellRanger. Assuming a doublet rate of 5%, 12,284 nuclei were removed using scDblFinder. Nuclei were excluded from downstream analysis if they had < 550 or > 5,000 features; < 1,000 or > 30,000 counts; or > 10% mitochondrial unique molecular identifiers. SCTransform was used to normalize the filtered data. Cluster identification was achieved with FindNeighbors, FindClusters, and RunUMAP. A resolution of 0.1 was used in FindClusters. The data were then logarithmically normalized using NormalizeData in which the assay type was set to “RNA” to visualize transcript(s) expression via DotPlot. Data were then passed through FindVariableFeatures and ScaleData to identify any outliers as well as scale and center features. All the above functions were used with default parameters. FindAllMarkers was used with assay set to “RNA” to identify differentially expressed transcripts between the 4 clusters. Differential expression analysis within a cluster and between 2 specific treatment groups was performed by first subsetting the data and then using FindMarkers with a logfc.threshold = 0.25. All treatment groups were compared with untreated *Col3a1^G938D/+^* mice, and differentially expressed transcripts were reported with corresponding Log_2_FC and adjusted *P* value (Wilcoxon ranked-sum test).

### Passive mechanical testing.

Intact rib cages with descending thoracic aortas from spironolactone-treated (P21–P60) VEDS mice and untreated controls were stored in PBS and shipped in dry-ice overnight to Northeastern University. Testing was performed according to established protocols (57) using a custom computer-controlled device. Aortas were subjected to pressurization cycles (10–140 mmHg) while axially extended at the crossover stretch or ± 5% thereof, and axial extension cycles to reach axial forces between 0 and 4 g while pressurized at 10, 60, 100, and 140 mmHg. Luminal pressure, outer diameter, axial stretch, and axial force were recorded. Unloaded thickness was measured on rings excised after testing. Estimated parameters for a 4-fiber family constitutive model were used to predict stretch, stress, and stiffness at 120 mmHg luminal pressure and crossover axial stretch, alongside cyclic distensibility between 80 and 120 mmHg. Data from male and female samples were combined based on published evidence of no sex-dependent differences in geometry-independent mechanical response (e.g., stretch, stress, stiffness, energy, distensibility) of the descending thoracic aorta in young mice (58).

### Code availability.

R scripts used for the snRNA-seq analysis are accessible through the GitHub repository at https://gist.github.com/LedaRestrepo/3a3f0d0db62e32f2d1d0db76a785cbee Code for ATAC-seq data analysis can be accessed through the GitHub repository at https://gist.github.com/LedaRestrepo/3a3f0d0db62e32f2d1d0db76a785cbee

### Statistics.

Analyses were performed using GraphPad Prism 10.4.2. Kaplan-Meier survival curves were compared using the Log-rank (Mantel-Cox) test. Deaths were recorded when vascular events were confirmed by hemothorax/hemoperitoneum at necropsy; mice euthanized for malocclusion, fight wounds, or genital prolapse were censored. Nonsurvival data are presented as mean ± SEM. Outliers were removed using the ROUT test (Q = 2% for qPCR, PSR, elastin, testosterone; Q = 1% for blood pressure). Normality was tested with Kolmogorov-Smirnov (if *n* ≥ 5 all groups) or Shapiro-Wilk (if *n* < 5 for any group). Normal data (≥ 3 groups) were examined using 1- or 2-way ANOVA with Šídák’s or Tukey’s post hoc test, as indicated in figure legends. Nonnormal data (≥ 3 groups) were examined using Kruskal-Wallis with Dunn’s post hoc test. Data generated by mechanical testing was examined with the nonparametric Mann-Whitney *U* test.

### Study approval.

All animal experiments were approved by the Johns Hopkins IACUC and were performed with adherence to their guidelines.

### Data availability.

The data that support the findings of this study are available in the main text or the supplemental materials; values for all data points in graphs are reported in the Supporting Data Values file. FASTQ files for bulk RNA-seq, snRNA-seq, and ATAC-seq are available in the Gene Expression Omnibus (GEO) repository under accession nos. GSE297353, GSE288058, and GSE315744.

## Author contributions

EEJ performed the majority of experiments, analyzed bulk-RNA-seq and snRNA-seq data, and wrote the first draft of the manuscript. CJB generated mouse models used for the study, established the rationale for the work, and assisted with data analysis and interpretation. RE performed all histological analyses and related quantification. LR performed and analyzed ATAC-seq data with code generated by DTS and MAB, who also oversaw the analysis. SL performed and analyzed all passive biomechanical experiments under supervision and guidance by CB. CAP provided bioinformatics support and assisted with ELISA experiments. AZ performed qPCR experiments. Both CAP and AZ contributed to data interpretation. MMB performed and analyzed immunoblots. OERG and EEB assisted with analysis of bulk RNA-seq and snRNA-seq data. OERG also assisted with drafting of the manuscript and data interpretation. HCD secured funding. HCD and EGM codirected the study, oversaw experimental design and planning, supervised data interpretation, and wrote and edited the manuscript with input from all authors. All authors reviewed and approved the final manuscript.

## Conflict of interest

CJB and HCD have submitted patent applications for pharmacological treatment of vascular Ehlers-Danlos syndrome (US Patent 11938135, US Patent Application 18/024,662).

## Funding support

This work is the result of NIH funding, in whole or in part, and is subject to the NIH Public Access Policy. Through acceptance of this federal funding, the NIH has been given a right to make the work publicly available in PubMed Central.

- DEFY Foundation to EEJ and HCD.
- Daskal Family Foundations to HCD.
- Marfan Foundation to EGM.
- NIH (1R01HL168473-01 to CB).

## Supplementary Material

Supplemental data

Unedited blot and gel images

Supplemental table 1

Supplemental table 2

Supplemental table 3

Supporting data values

## Version 1 04/28/2026

In-Press Preview

## Version 2 06/22/2026

Electronic publication
