# Supplementary material for: Steroid hormone antagonism affords vascular protection in a mouse model of vascular Ehlers-Danlos syndrome
Source: JCI Insight. 2026 Apr 28;11(12):e198202. doi: 10.1172/jci.insight.198202 (PMC13313488; doi:10.1172/jci.insight.198202)
Supplement: Supplemental data [file jciinsight-11-198202-s310.pdf]

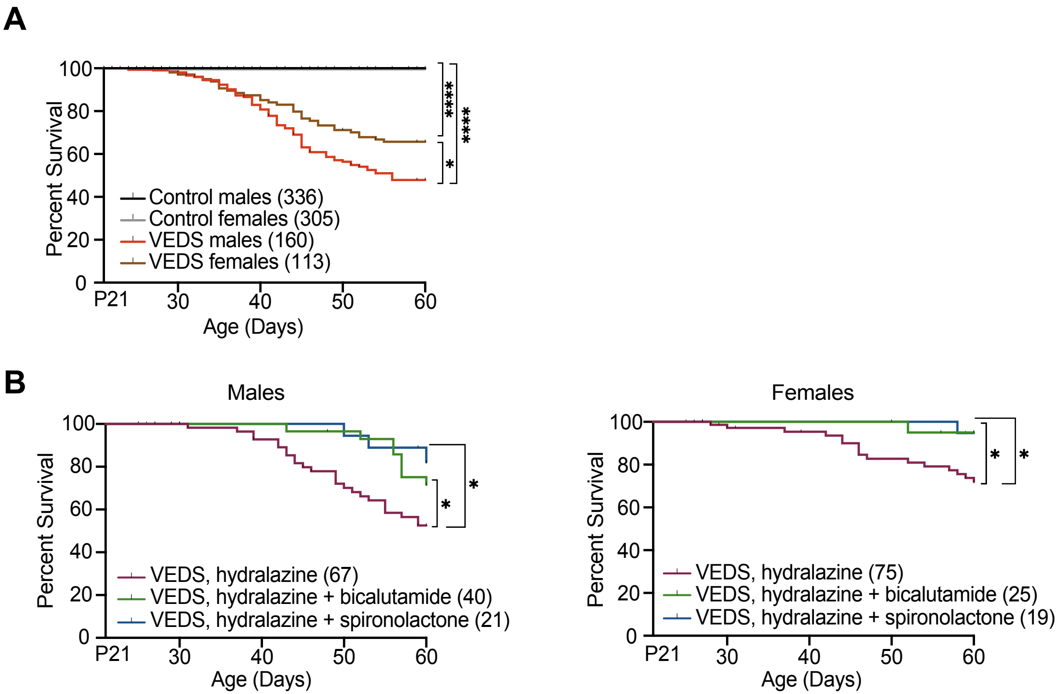

Supplemental Figure 1. The risk of death by aortic rupture is sexually dimorphic in VEDS mice.
(**A**) Kaplan-Meier survival curve comparing male control (N = 336) and VEDS mice (N = 160) to female control (N = 305) and VEDS mice (N = 113). (**B**) Kaplan-Meier survival curve comparing VEDS mice treated with hydralazine starting at birth (males N = 67, females N = 75) and VEDS mice that were also treated with bicalutamide (males N = 40, females N = 25) or spironolactone (males N = 21, females N = 19) starting at P21. P-values refer to Log-rank Mantel-Cox test; * = P ≤ 0.05 and **** = P ≤ 0.0001.

A

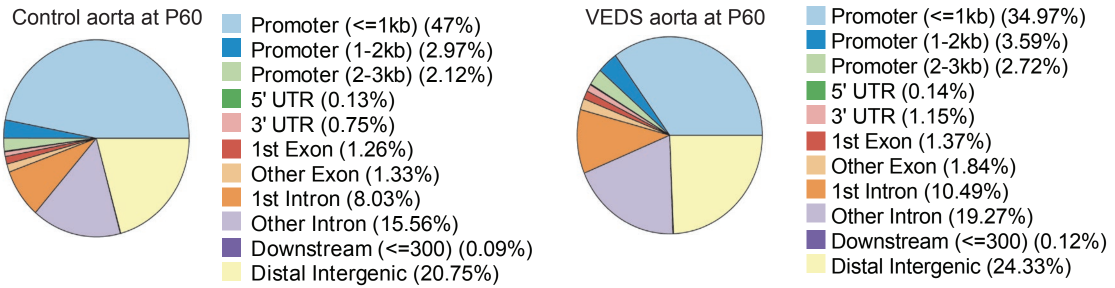

B

Motifs enriched in accessible peaks in control aorta at P60

N	Motif	R	W	Z	I
1	MEF2C	0.97	1.00	7.72	0.85
2	FOSL2:JUN	0.97	0.81	7.25	1.00
3	CTCF	0.96	0.40	6.60	0.69
4	NFIX	0.92	0.53	5.36	0.24
5	ATF2	0.93	0.70	5.03	0.50
6	NF1:FOXA1	0.94	0.39	4.83	0.24
7	MEF2C	0.80	0.74	3.84	0.23
8	TEAD	0.93	0.59	3.72	0.30
9	SRF	0.89	0.64	3.48	0.29
10	NFIC	0.90	0.64	3.01	0.25
11	V_GC_01	0.93	0.60	2.97	0.23
12	V_FOXO1_01	0.65	0.87	2.39	0.26
13	NR3C1	0.85	0.46	1.89	0.10
14	TLX	0.68	0.74	1.52	0.16
15	V_HSF1_01	0.64	0.74	1.47	0.18
16	CTCFL	0.53	0.65	1.43	0.13
17	NKX2.5	0.71	0.69	1.10	0.12
18	EBF1	0.76	0.95	1.08	0.20
19	THAP11	0.74	0.91	1.07	0.18
20	V_ATF6_01	0.55	0.81	0.98	0.21
21	DUX	0.67	0.89	0.41	0.12
22	FOXE1	0.57	-1.00	-1.12	0.24
23	V_GATA2_01	0.62	-0.68	-1.30	0.13
24	ZBTB7B_second	0.70	-1.40	-1.80	0.27
25	FOXL1	0.59	-1.09	-1.82	0.28
26	UA3	0.78	-1.27	-2.29	0.43
27	ZFP105_primary	0.75	-1.56	-2.48	0.68
28	ETV4	0.71	-1.11	-2.60	0.40
29	PIT1	0.62	-0.86	-2.98	0.25
30	ZEB1	0.86	-1.50	-4.04	0.89

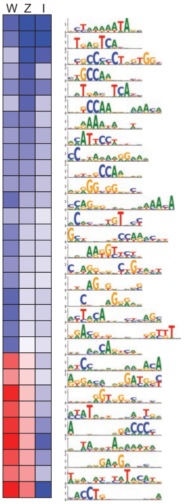

Motifs enriched in accessible peaks in VEDS aorta at P60

N	Motif	R	W	Z	I
1	FOSL1:JUND	0.98	0.49	7.07	0.92
2	MEF2C	0.94	0.56	6.68	0.37
3	CTCF	0.96	0.12	6.34	0.21
4	CTCF	0.92	0.19	5.97	0.21
5	NFIC	0.93	0.56	5.93	0.46
6	V_RSRFC4_01	0.88	0.32	5.39	0.27
7	JUND (var.2)	0.93	0.43	4.61	0.42
8	MEF2C	0.83	0.31	3.99	0.17
9	TEAD3	0.95	0.90	3.74	0.47
10	NF1	0.87	0.48	3.31	0.35
11	NF1:FOXA1	0.94	0.32	3.12	0.23
12	V_GC_01	0.92	0.40	2.83	0.24
13	SRF_primary	0.92	0.59	2.74	0.40
14	RFX5	0.93	0.38	2.67	0.21
15	PGR	0.78	0.77	2.29	0.28
16	V_CAP_01	0.74	0.75	1.87	0.32
17	CTCF	0.55	0.50	1.76	0.19
18	ZNF317	0.54	0.86	1.54	0.25
19	HIC1	0.63	0.48	1.37	0.17
20	ETV4	0.83	0.67	1.09	0.26
21	V_AP1FJ_01	0.78	1.00	0.33	0.30
22	V_IRF2_01	0.68	0.95	-0.22	0.28
23	ZEB1	0.68	-0.41	-1.01	0.15
24	GATA1:TAL1	0.63	-0.69	-1.23	0.29
25	GATA2	0.60	-0.60	-1.44	0.21
26	PAX7	0.60	-1.17	-1.69	0.94
27	YY1	0.55	-1.06	-1.99	0.68
28	UA3	0.82	-1.41	-2.44	0.62
29	ETV4	0.68	-0.88	-3.20	0.49
30	ZEB1	0.94	-0.86	-5.31	1.00

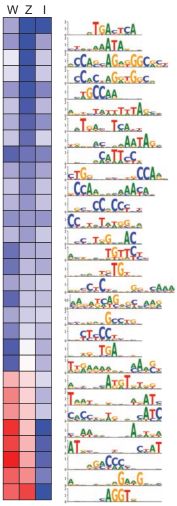

Supplemental Figure 2. Control and VEDS aorta show similar chromatin accessibility profiles and motif enrichment. (**A**) Distribution of pseudo bulk-ATAC peak locations for control (23,507 peaks) and VEDS (19,306 peaks). Categories: promoter (≤1 kb, 1–2 kb, 2–3 kb), 5′/3′UTR, 1st/other exon, 1st/other intron, downstream (≤3 kb), and distal intergenic. (**B**) Top motif families and logos preferentially enriched in accessible chromatin regions in control and VEDS aorta. Heatmap shows Redundancy (R), Weight (W), Z-score (Z), and Importance (I). Position weight matrices (PWM) learned de novo from gkm-SVM weight vectors using gkmPWM algorithm with 300-bp summit-centered windows (l=11, k=7). R = maximum Pearson correlation to other PWMs; W = gkmPWM model contribution score; Z = standardized mean of highest-weighted gkm; I = relative model error increase when removing that PWM.

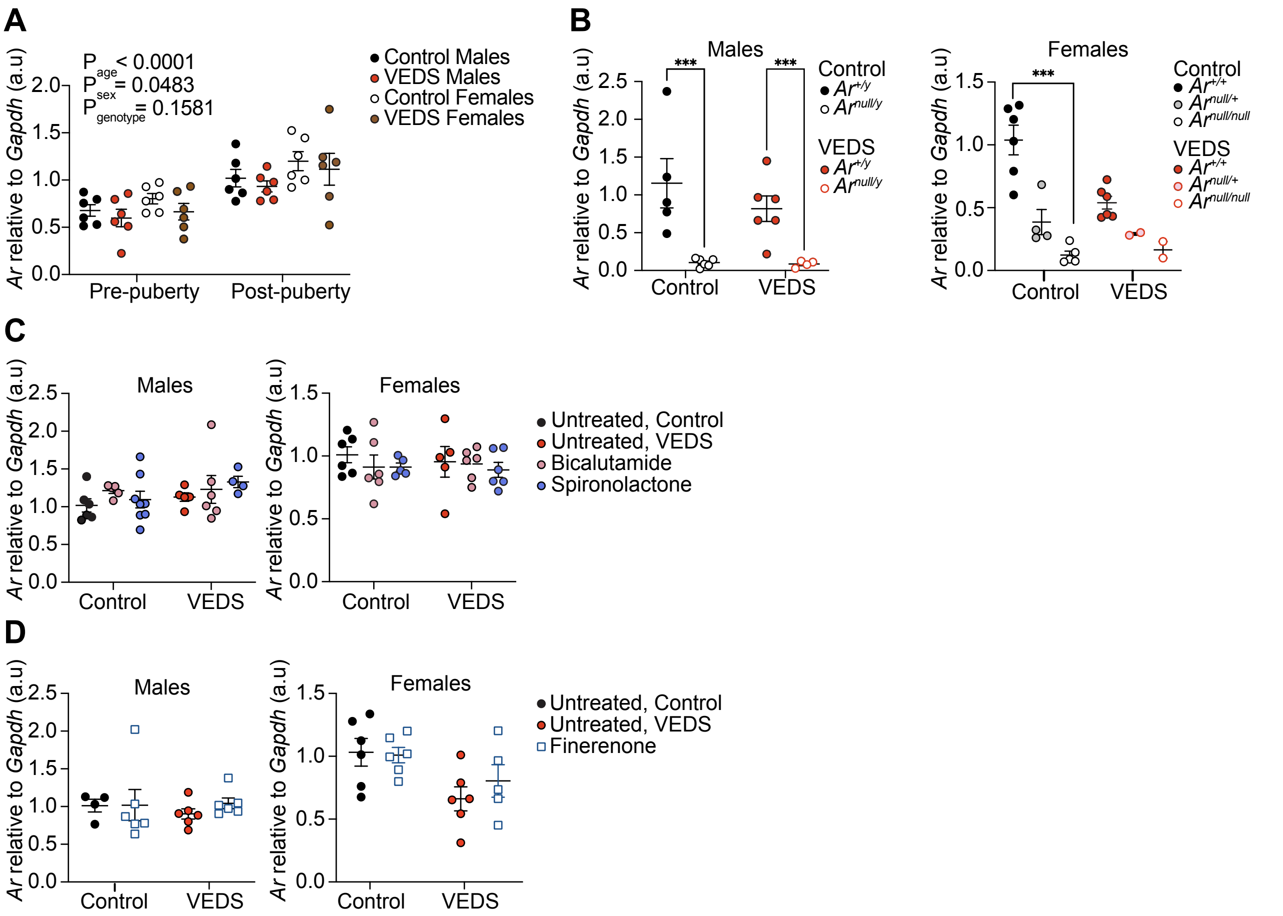

Supplemental Figure 3. Effect of AR/MR antagonism on *Ar* expression in the descending thoracic aorta.
(**A**) *Ar* expression (qPCR, relative to *Gapdh*) at P30 and P60 in male and female control and VEDS mice, normalized to post-puberty control males (N = 6 per group). P-values refer to three-way ANOVA with Tukey's post hoc test. (**B**) *Ar* expression in adult mice (qPCR, relative to *Gapdh*), normalized to control $Ar^{+/y}$ (males) or $Ar^{+/+}$ (females). Males: control $Ar^{+/y}$ (N = 6), control $Ar^{null/y}$ (N = 4), VEDS $Ar^{+/y}$ (N = 6), VEDS $Ar^{null/y}$ (N = 4); P-values refer to two-way ANOVA with Dunn-Šidák post hoc test; *** = P ≤ 0.001. Females: control $Ar^{+/+}$ (N = 6), control $Ar^{null/+}$ (N = 4), control $Ar^{null/null}$ (N = 4), VEDS $Ar^{+/+}$ (N = 6), VEDS $Ar^{null/+}$ (N = 2), VEDS $Ar^{null/null}$ (N = 2); P-values refer to Kruskal-Wallis with Dunn's post hoc test; *** = P ≤ 0.001. (**C**) *Ar* expression in adult mice (qPCR, relative to *Gapdh*), normalized to sex-matched untreated controls. Males: N = 6 except spironolactone-treated controls (N = 8), untreated VEDS (N = 5), spironolactone-treated VEDS (N = 4). Females: N = 6 except spironolactone-treated controls (N = 5), untreated VEDS (N = 5). No significant differences noted via two-way ANOVA with Dunn-Šidák post hoc test. (**D**) *Ar* expression in adult mice (qPCR, relative to *Gapdh*), normalized to sex-matched untreated controls (N = 6 except untreated control males N = 4, finerenone-treated females N = 5). No significant differences noted via Kruskal-Wallis with Dunn's post hoc test (males) or two-way ANOVA with Dunn-Šidák post hoc test (females). All samples were assessed at P60. For all panels, each symbol represents an independent biological sample, the horizontal bar represents the median value, and the error bars represent the standard error of the mean.

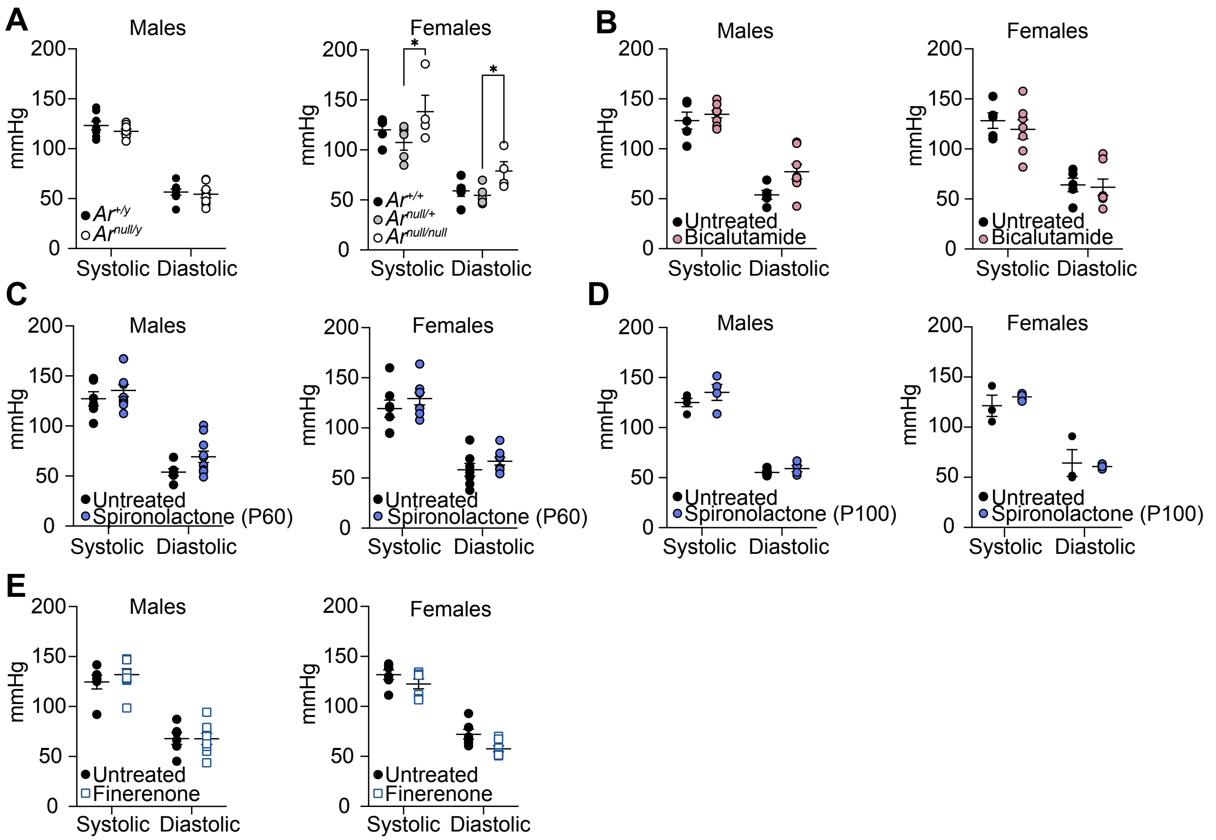

Supplemental Figure 4. Effect of AR or MR antagonism on hemodynamic parameters in control *Col3a1*$^{+/+}$ mice.
(**A**) Blood pressure measurements for *Ar*$^{+/y}$ (N = 8) and *Ar*$^{null/y}$ male mice (N = 8), and *Ar*$^{+/+}$ (N = 5), *Ar*$^{null/+}$ (N = 6), and *Ar*$^{null/null}$ female mice (N = 4). (**B**) Blood pressure measurements of untreated and bicalutamide-treated male and female mice (P21 to P60) mice, N = 5 for all groups. (**C**) Blood pressure measurements comparing untreated mice (males N = 6, females N = 7) to spironolactone-treated (P21 to P60) mice (males N = 10, females N = 8). (**D**) Blood pressure measurements comparing untreated mice (males N = 4, females N = 3) to mice treated with spironolactone from P60 to P100 (males N = 4, females N = 3). (**E**) Blood pressure measurements comparing untreated mice (males N = 6, females N = 6) to mice treated with finerenone from P21 to P60 (males N = 8, females N = 7). Blood pressures were measured at P60 for panels (A), (B), (C), and (E) and at P100 for (D). Significance was assessed with two-way ANOVA followed by Dunn-Šidák post-hoc test for all panels except females in panel (B) which used a Kruskal-Wallis test with Dunn's multiple comparisons post hoc test; * = $P \leq 0.05$. For all panels, each symbol represents an independent biological sample, the horizontal bar represents the median value, and the error bars represent the standard error of the mean.

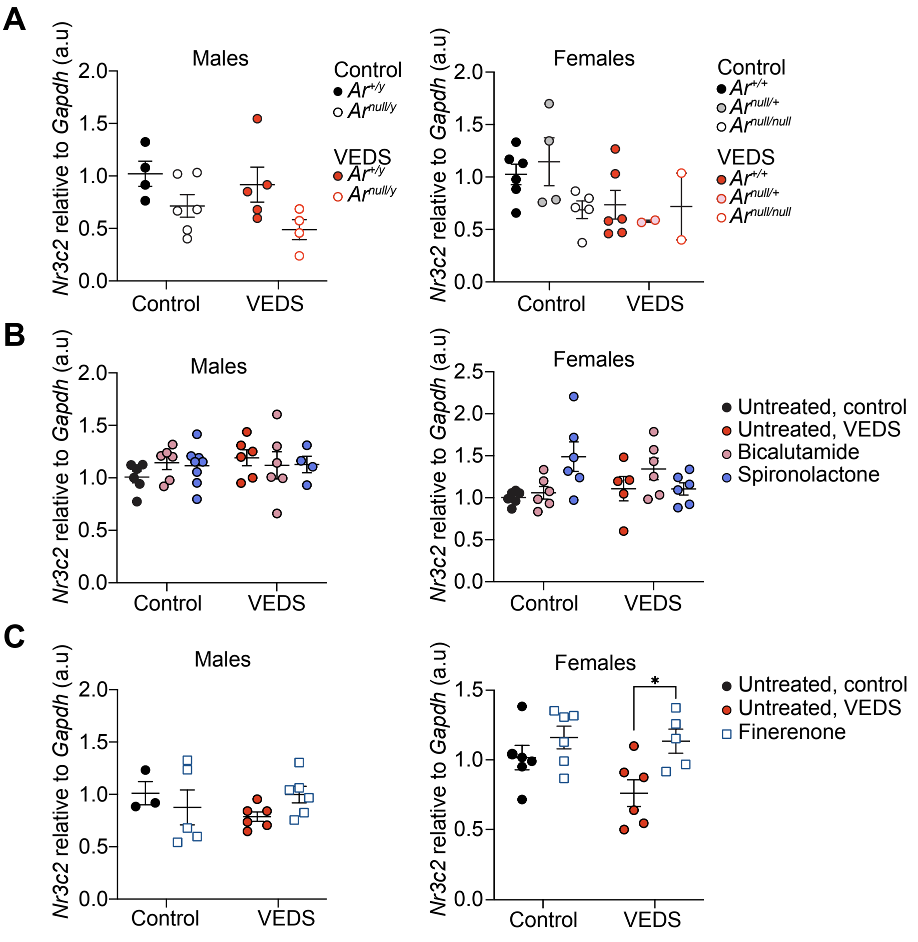

Supplemental Figure 5. Effect of pharmacological AR/MR on MR expression in the descending thoracic aorta.
(**A**) *Nr3c2* expression (qPCR, normalized to *Gapdh*) in the descending thoracic aorta of adult mice. Controls (males, N = 6; females, N = 6), control $Ar^{null/y}$ (males N = 4), VEDS (males, N = 6; females, N = 6), VEDS $Ar^{null/y}$ (males, N = 6), control $Ar^{null/+}$ (females, N = 4), control $Ar^{null/null}$ (females, N = 4), VEDS $Ar^{null/+}$ (females, N = 2), VEDS $Ar^{null/null}$ (females, N = 2). (**B**) *Nr3c2* expression in adult mice by genotype and treatment (N = 6 except for spironolactone-treated control males N = 8, spironolactone-treated VEDS males N = 4). (**C**) *Nr3c2* expression in adult mice treated with finerenone or untreated. Untreated controls (males, N=3; females, N = 6), finerenone-treated controls (males, N = 5; females, N = 6), untreated VEDS (males, N = 6; females, N = 5), finerenone-treated VEDS (males, N = 6; females, N = 5). Data are normalized to the median value of sex-matched untreated controls. Significant differences were calculated using two-way ANOVA followed by Dunn-Šidák post-hoc test for all panels except females in panel (A) which used a Kruskal-Wallis test with Dunn's multiple comparisons post-hoc test; * = P ≤ 0.05. All samples were assessed at P60. For all panels, each symbol represents an independent biological sample, the horizontal bar represents the median value, and the error bars represent the standard error of the mean.

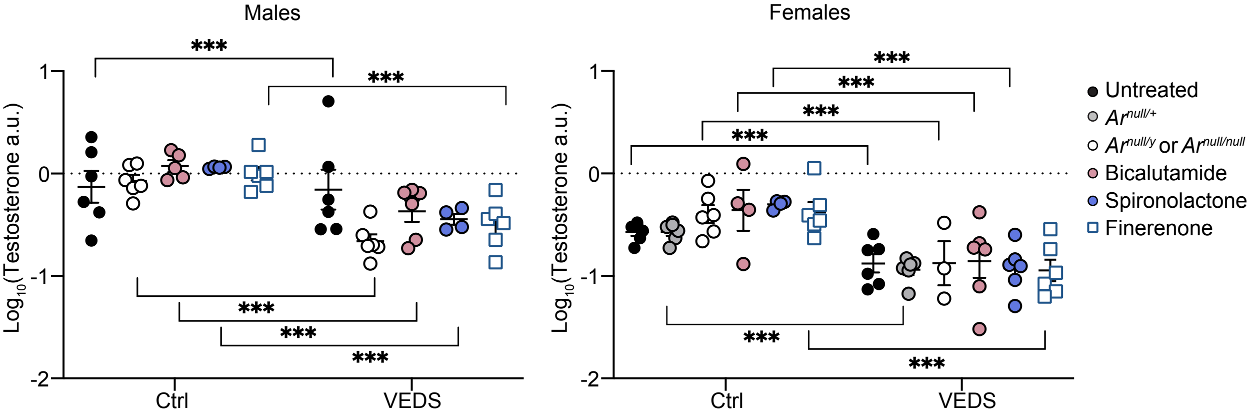

Supplemental Figure 6. Testosterone decreases with genotype but not treatment regimen.
Testosterone concentration as assessed by ELISA in serum from adult male and female mice normalized to the mean of untreated control (Ctrl) male samples. Male samples: N = 6 for all groups except untreated (N = 5), bicalutamide-treated (N = 5), and spironolactone-treated (N = 4) controls, untreated VEDS (N = 5), and spironolactone-treated VEDS (N = 4). Female samples: N = 6 for all groups except untreated (N = 5), bicalutamide-treated (N = 3), and spironolactone-treated (N = 4) controls, and VEDS $Ar^{null/null}$ (N = 3). Significant differences were assessed using two-way ANOVA with Tukey's multiple comparisons post-hoc test; *** = P ≤ 0.001. Each symbol represents an independent biological sample, the horizontal bar represents the median value, and the error bars represent the standard error of the mean.

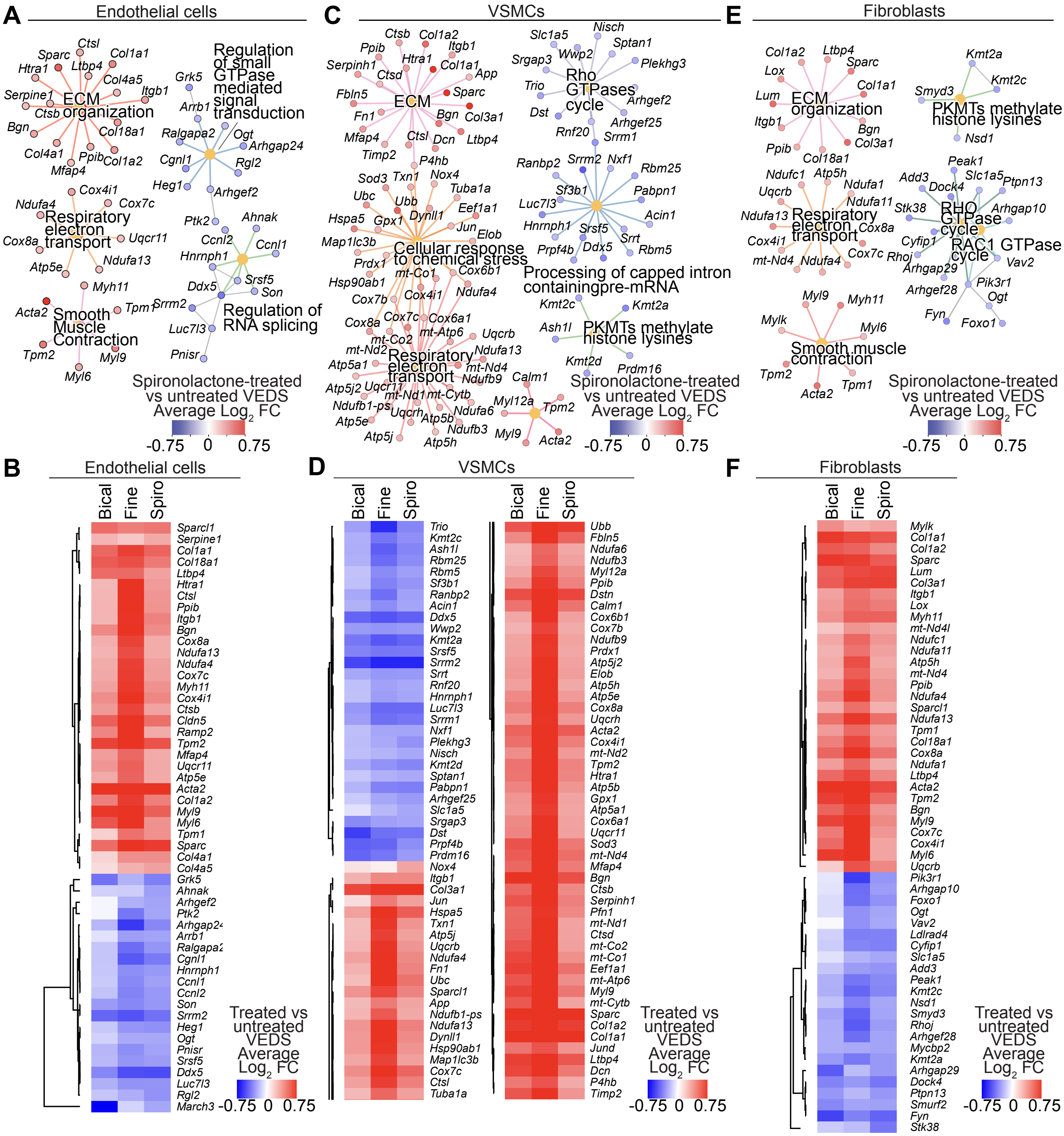

Supplemental Figure 7. Enriched terms and transcripts modulated in a similar manner by spironolactone, finerenone, and/or bicalutamide in major aortic cell types.
Enriched terms for transcripts similarly modulated by isolated or dual AR/MR inhibition (<20% difference in spironolactone effect vs. other treatments) in endothelial cells (**A**, **B**), VSMCs (**C**, **D**), and fibroblasts (**E**, **F**). Network nodes (A, C, E) colored by expression change in spironolactone-treated vs. untreated VEDS aorta. Heatmaps (B, D, F) show expression in spironolactone (Spiro)-, finerenone (Fine)-, and bicalutamide (Bical)-treated samples relative to untreated VEDS aorta. Red = upregulated; blue = downregulated.

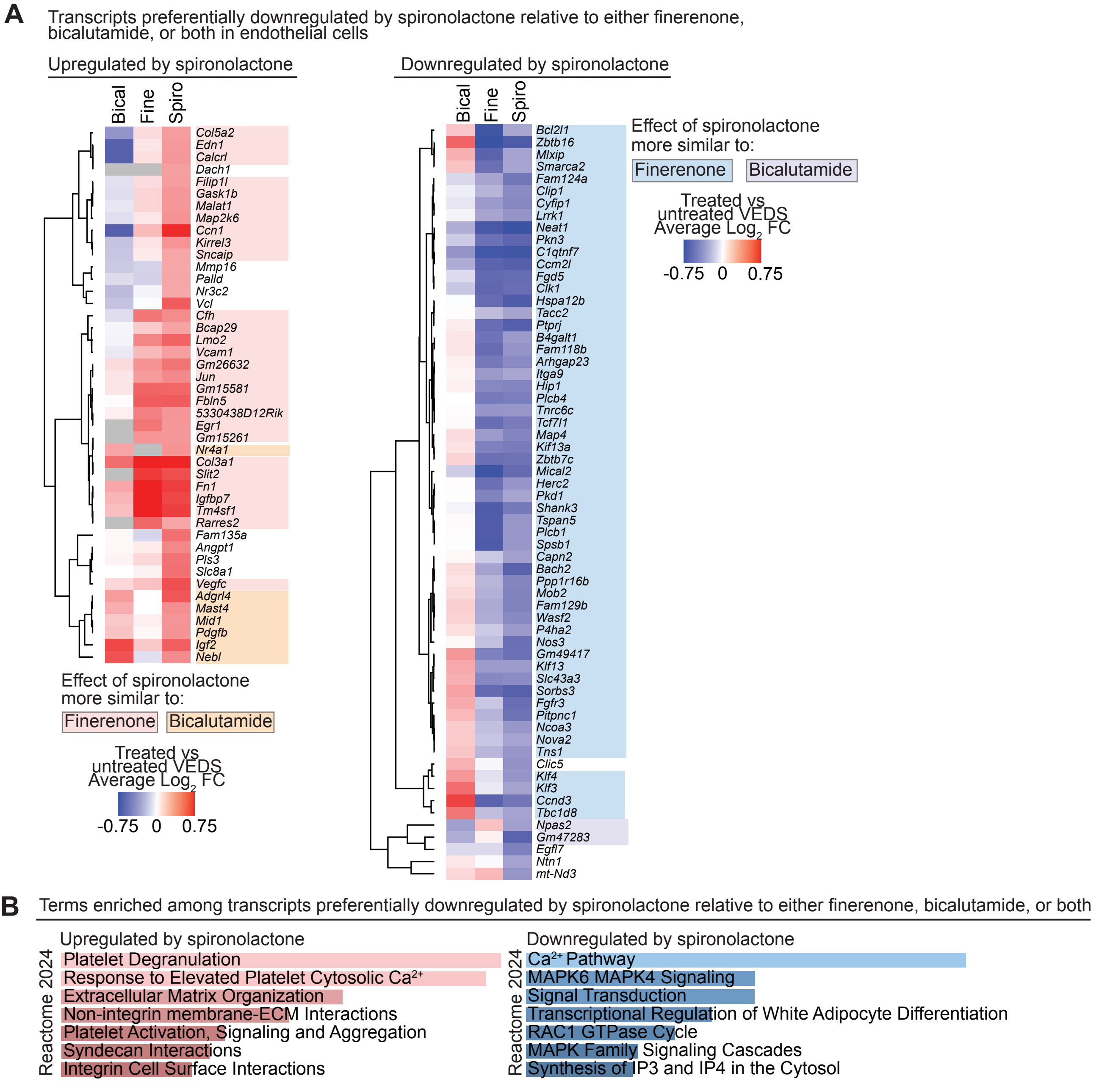

Supplemental Figure 8. Transcripts and enrichment for transcripts preferentially modulated by spironolactone relative to finerenone, bicalutamide, or both in endothelial cells.
(**A**) Hierarchical clustering and heatmap for transcripts preferentially modulated by spironolactone vs. finerenone, bicalutamide, or both in endothelial cells. Expression shown in spironolactone (Spiro)-, finerenone (Fine)-, and bicalutamide (Bical)-treated samples relative to untreated VEDS aorta. Red = upregulated; blue = downregulated. (**B**) Enriched terms for up- and downregulated transcripts from A (Enrichr). Shading inversely proportional to enrichment P-value.

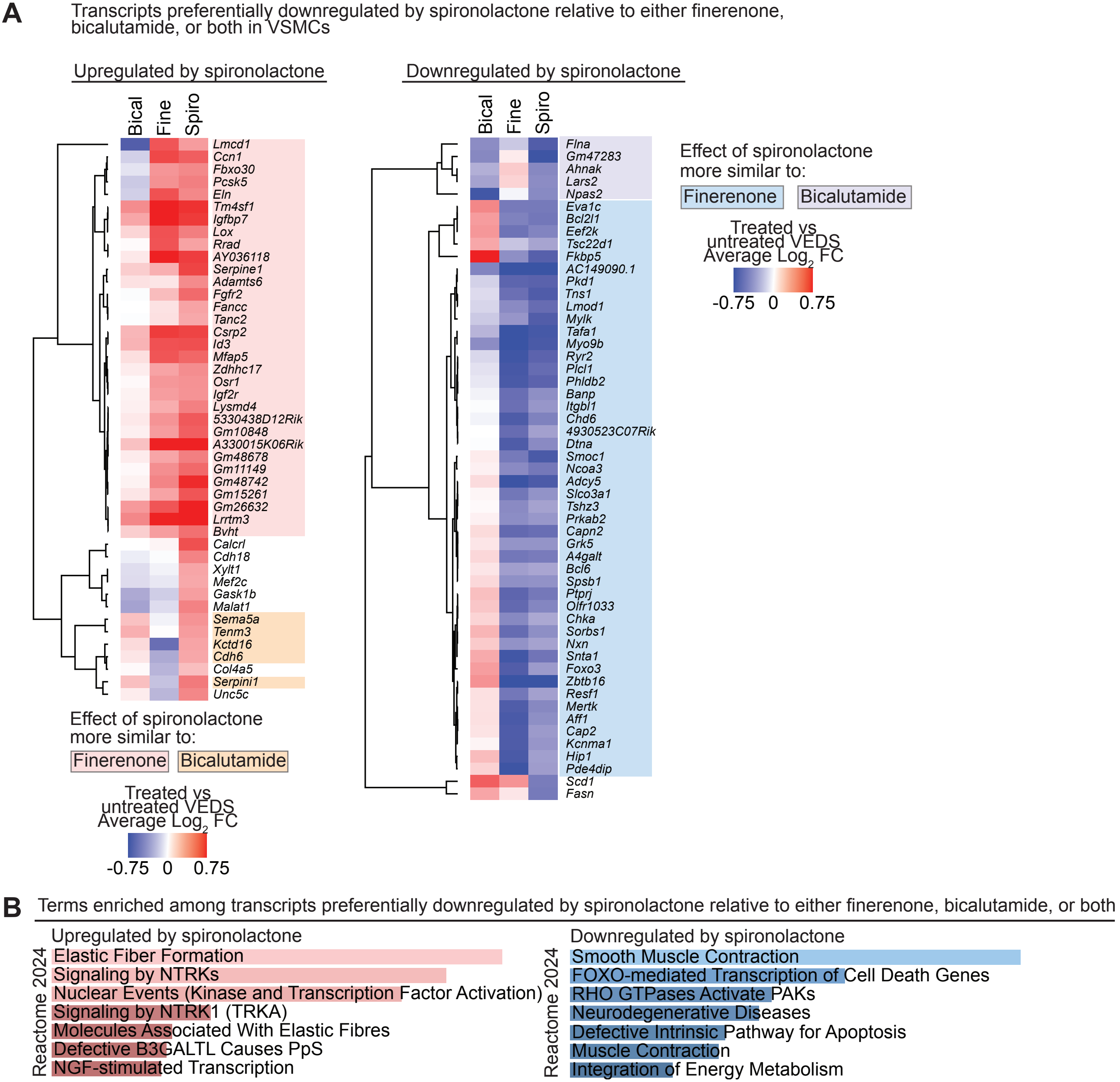

Supplemental Figure 9. Transcripts and enrichment for transcripts preferentially modulated by spironolactone relative to finerenone, bicalutamide, or both in VSMCs.
(**A**) Hierarchical clustering and heatmap for transcripts preferentially modulated by spironolactone vs. finerenone, bicalutamide, or both in VSMCs. Expression shown in spironolactone (Spiro)-, finerenone (Fine)-, and bicalutamide (Bical)-treated samples relative to untreated VEDS aorta. Red = upregulated; blue = downregulated. (**B**) Enriched terms for up- and downregulated transcripts from A (Enrichr). Shading inversely proportional to enrichment P-value.

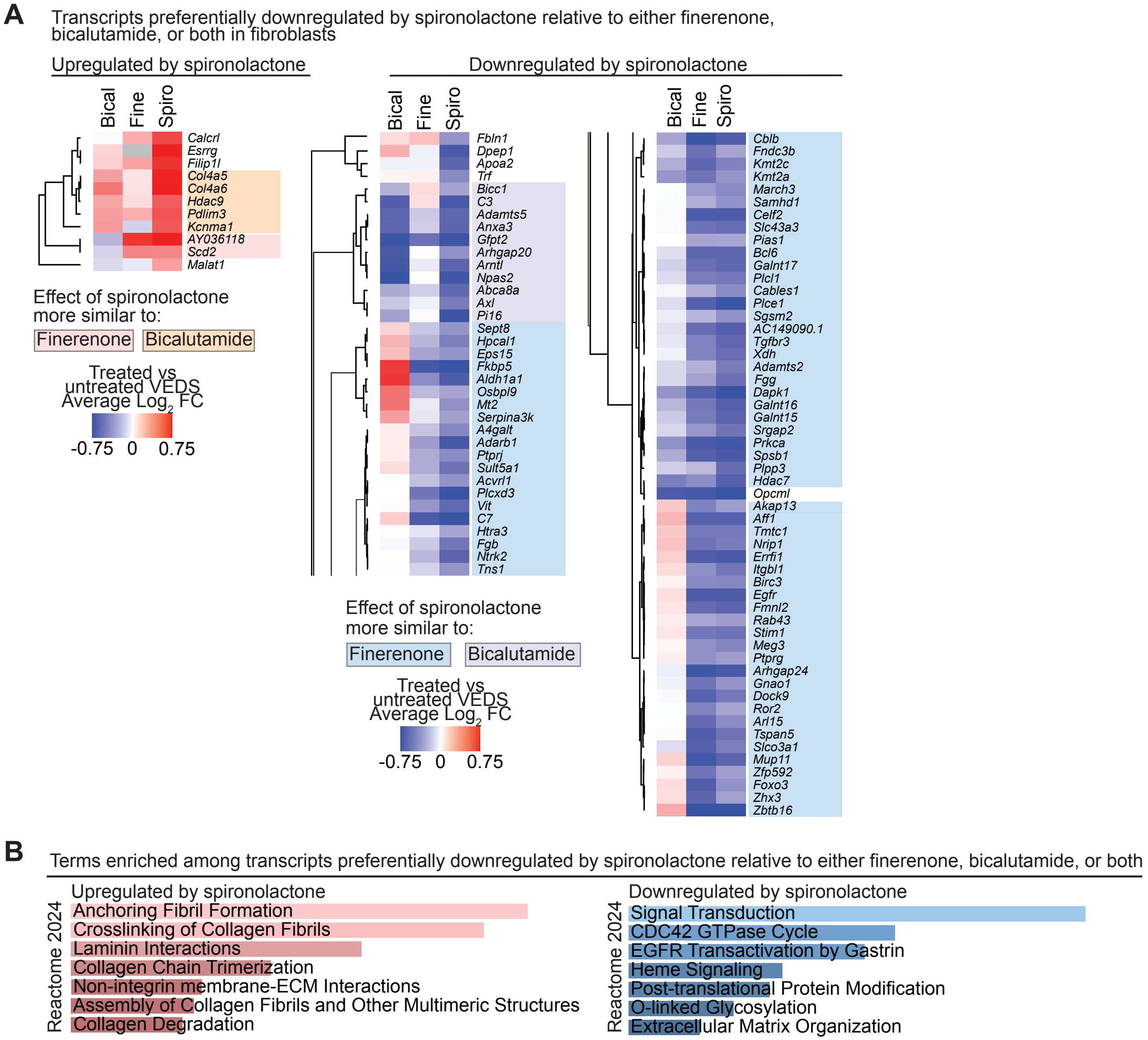

Supplemental Figure 10. Transcripts and enrichment for transcripts preferentially modulated by spironolactone relative to finerenone, bicalutamide, or both in fibroblasts.
(**A**) Hierarchical clustering and heatmap for transcripts preferentially modulated by spironolactone vs. finerenone, bicalutamide, or both in fibroblasts. Expression shown in spironolactone (Spiro)-, finerenone (Fine)-, and bicalutamide (Bical)-treated samples relative to untreated VEDS aorta. Red = upregulated; blue = downregulated. (**B**) Enriched terms for up- and downregulated transcripts from A (Enrichr). Shading inversely proportional to enrichment P-value.

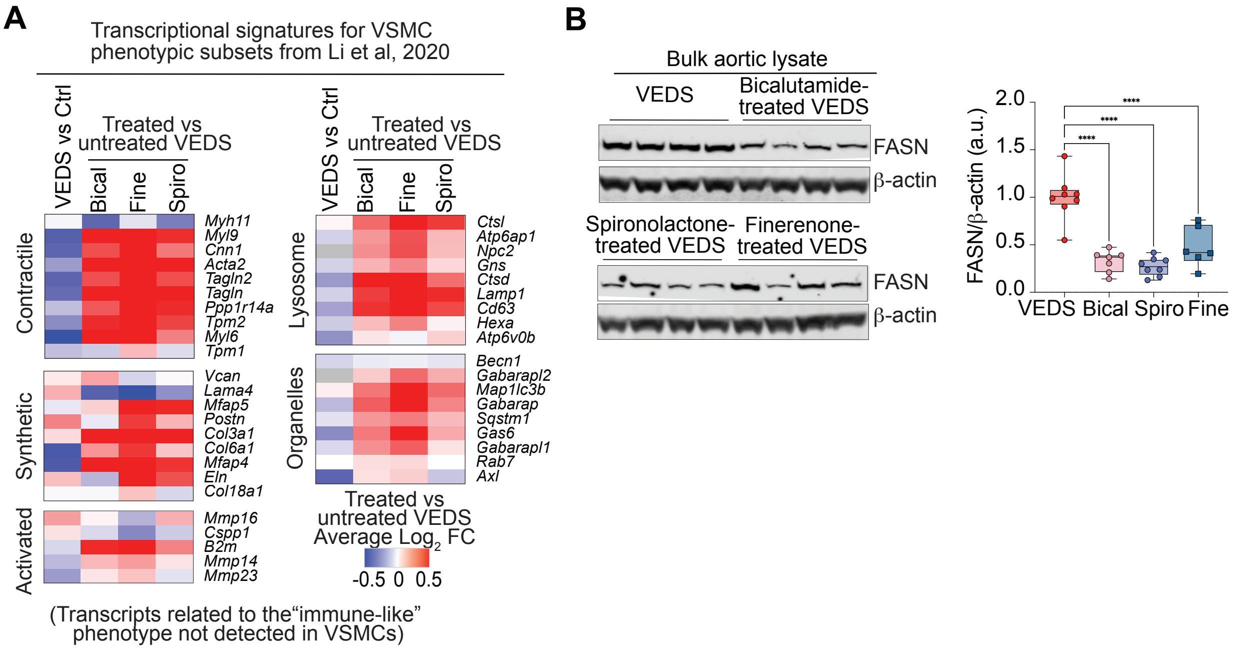

Supplemental Figure 11.

AR/MR antagonism promotes a synthetic VSMC phenotype and reduces levels of fatty acid synthase (FASN).

(**A**) Heatmap of VSMC phenotypic modulation transcripts in aneurysmal aorta (Li et al, 2020). Expression in VSMCs from VEDS vs. control aorta, and spironolactone (Spiro)-, finerenone (Fine)-, and bicalutamide (Bical)-treated vs. untreated VEDS mice. Red = upregulated; blue = downregulated. (**B**) Representative immunoblot and quantification of aortic lysates from untreated male VEDS and male VEDS mice treated with bicalutamide (N = 7), spironolactone (N = 8), or finerenone (N = 6) from P21-P60, probed for FASN and β-actin. Signal is normalized to the average of untreated VEDS, and reference samples across immunoblots; P-value refers to one-way ANOVA with Šidák post-hoc test; **** = $P \leq 0.0001$. Each symbol represents an independent biological sample, the horizontal bar represents the median value, and whiskers represent the range from minimum to maximum values.

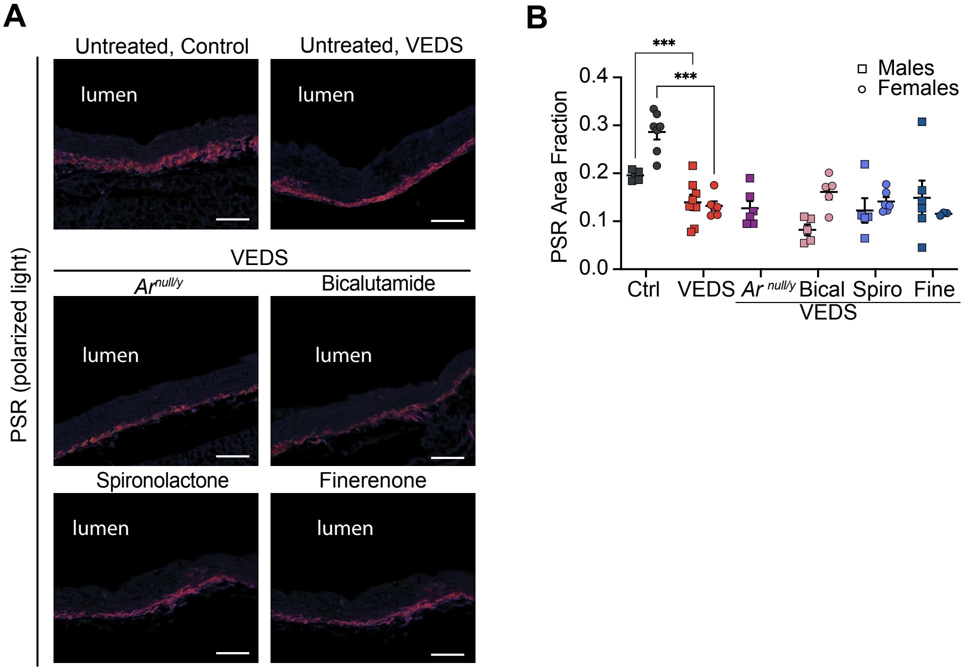

Supplemental Figure 12. AR/MR inhibition does not associate with increased collagen deposition in the adventitial layer. (**A**) Representative picrosirius red (PSR)-stained aortic sections from descending thoracic aorta by genotype and treatment. In polarized light, red represents collagen. Scale bar is 50 μm. (**B**) Quantification of PSR signal in aortic sections from control (males, N = 5; females, N = 7), VEDS (males, N = 8; females, N = 6), VEDS *Ar*$^{null/y}$ (males N = 6), bicalutamide-treated VEDS (males, N = 5; females, N = 5), spironolactone-treated VEDS (males, N = 5; females, N = 6), and finerenone-treated VEDS (males, N = 6; females N = 3) mice. P-values refer to two-way ANOVA followed by Šídák's multiple comparisons post-hoc test comparing treatments to sex-matched VEDS samples; *** = P ≤ 0.001. Each symbol represents an independent biological sample, the horizontal bar represents the median value, and the error bars represent the standard error of the mean.

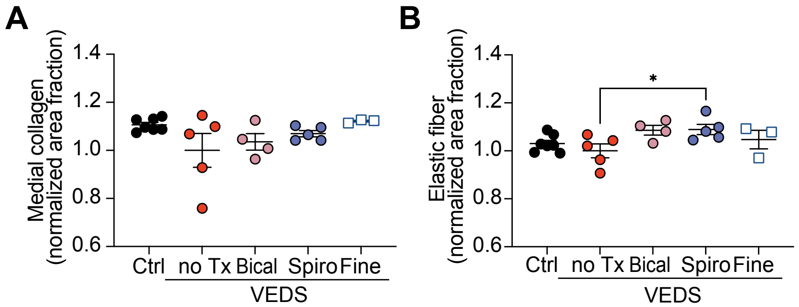

Supplemental Figure 13.

Effects of AR/MR antagonisms on medial collagen and elastin content in the aorta of female VEDS mice.
Quantification of medial collagen (**A**) and elastic fiber content (**B**) in the descending thoracic aorta of female untreated control (N = 7) and VEDS (N = 5) mice, and bicalutamide (Bical)- (N = 4), spironolactone (Spiro)- (N = 6), and finerenone (Fine)- (N = 6) treated female VEDS mice. Data are normalized to the average of untreated VEDS samples. P-value refers to one-way ANOVA with Šidák post-hoc test; * = P ≤ 0.05. Each symbol represents an independent biological sample, the horizontal bar represents the median value, and the error bars represent the standard error of the mean.

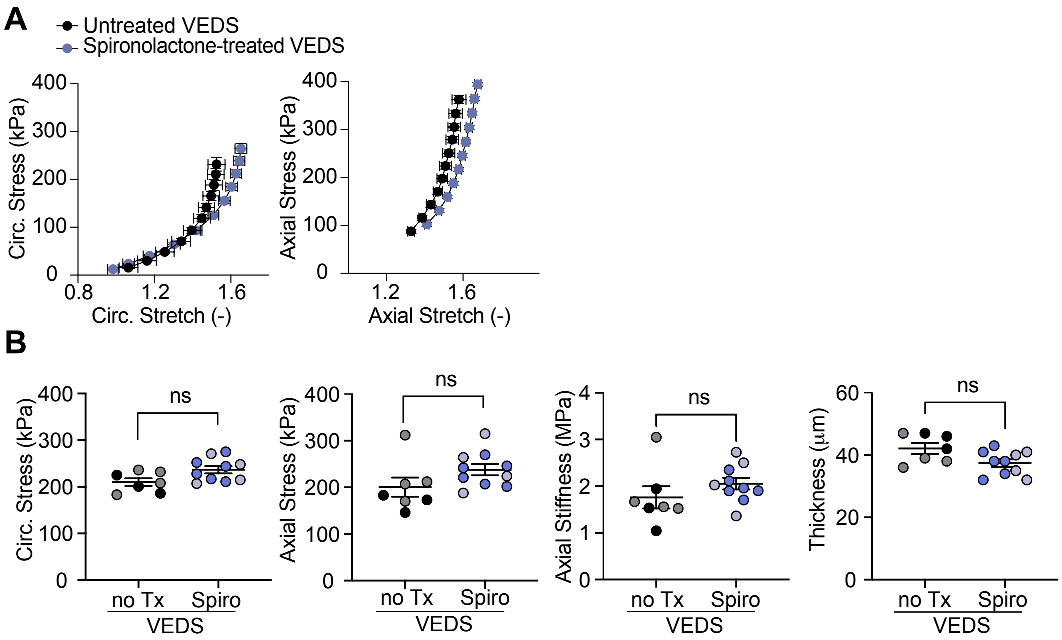

Supplemental Figure 14.

Spironolactone treatment renders the descending thoracic aorta of VEDS mice more deformable.

(**A**) Geometry-independent biaxial mechanical response of descending thoracic aortic tissues from VEDS mice treated with spironolactone from P21 to P60 (N = 10) or left untreated (N = 7). Spironolactone-treated aortas are more deformable in both the circumferential and axial directions across a broad, physiologically relevant stretch range, such that a greater stretch is required to achieve a given level of stress. (**B**) Aortic wall thickness, biaxial stress, and axial tissue stiffness under representative systolic pressure (120 mmHg) are not significantly affected by spironolactone treatment. Each symbol represents an individual biological replicate; lighter shading indicates female mice.
