# Supplementary figures and images for: Steroid hormone antagonism affords vascular protection in a mouse model of vascular Ehlers-Danlos syndrome
Source: JCI Insight. 2026 Apr 28;11(12):e198202. doi: 10.1172/jci.insight.198202 (PMC13313488; doi:10.1172/jci.insight.198202)

## Immunoblot in Sup. Figure 1

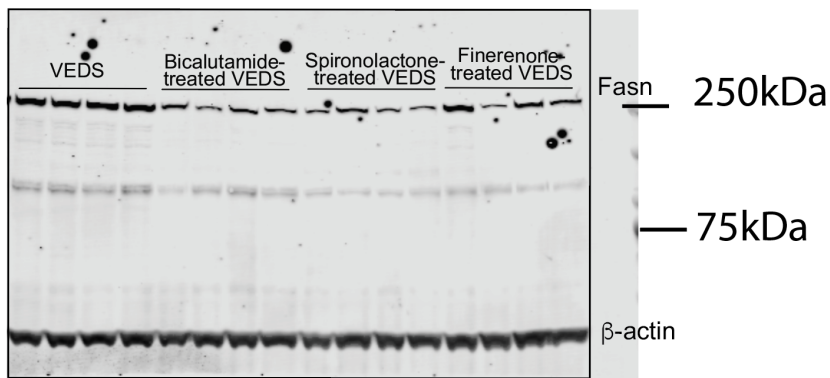

## Quantified in Sup. Fig. 1 but not shown

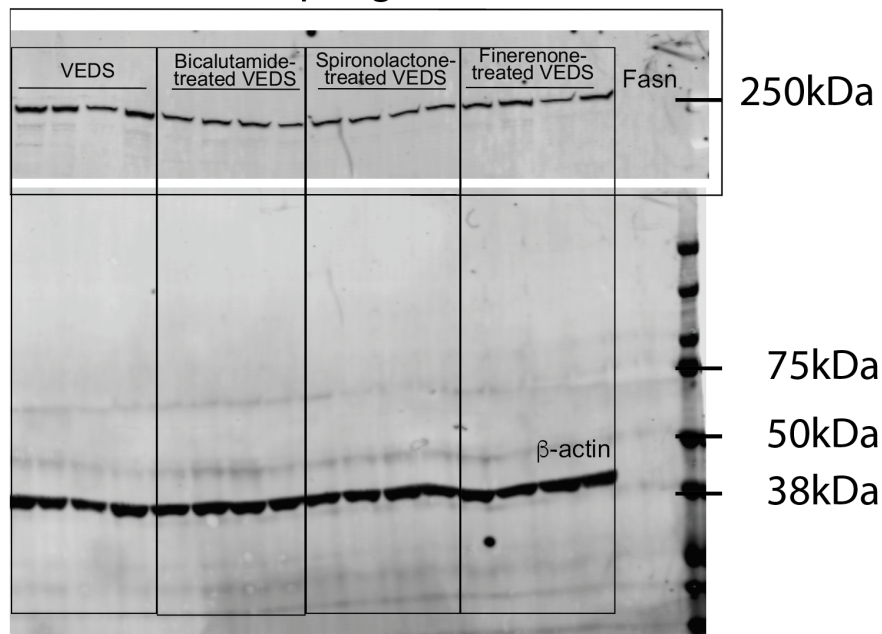

Supplement: Unedited blot and gel images [file jciinsight-11-198202-s311.pdf]
